# Management of Patients with Epithelial Ovarian Cancer: A Systematic Comparison of International Guidelines from Scientific Societies (AIOM-BGCS-ESGO-ESMO-JGSO-NCCN-NICE)

**DOI:** 10.3390/cancers17243915

**Published:** 2025-12-07

**Authors:** Martina Arcieri, Veronica Tius, Sara Filippin, Giovanni Aletti, Domenica Lorusso, Anna Fagotti, Jalid Sehouli, Ignacio Zapardiel, Pierandrea De Iaco, Paolo Scollo, Andrea Ciavattini, Marco Petrillo, Violante Di Donato, Federica Perelli, Giorgio Bogani, Stefano Restaino, Giuseppe Vizzielli, the Gynecologic Oncology Group

**Affiliations:** 1 Clinic of Obstetrics and Gynecology, “S. Maria della Misericordia” University Hospital, Azienda Sanitaria Universitaria Friuli Centrale (ASUFC), 33100 Udine, Italy; martina.arcieri@asufc.sanita.fvg.it (M.A.);; 2 Medical Area Department (DAME), in Department of Medicine (DMED), University of Udine, 33100 Udine, Italy; veronica.tius@asufc.sanita.fvg.it (V.T.);; 3 Division of Gynecologic Oncology, European Institute of Oncology (IEO), IRCCS Milan, 20141 Milan, Italy; 4 Unit of Gynecologic Oncology, IRCCS Humanitas Research Hospital, Rozzano, 20089 Milan, Italy; 5 Gynecologic Oncology Unit, Fondazione Policlinico Universitario A Gemelli IRCCS, 00168 Rome, Italy; 6 Department of Gynecology with Center for Oncological Surgery, Campus Virchow Klinikum, Charité-Universitätsmedizin Berlin, 13353 Berlin, Germany; 7 Gynecologic Oncology Unit, La Paz University Hospital, 28046 Madrid, Spain; 8 IRCCS Azienda Ospedaliero-Universitaria di Bologna, 40138 Bologna, Italy; 9 Department of Medical and Surgical Sciences (DIMEC), University of Bologna, 40126 Bologna, Italy; 10 Division of Gynecology and Obstetrics, Cannizzaro Hospital, 95021 Catania, Italy; 11 Faculty of Medicine and Surgery, Kore University, 94100 Enna, Italy; 12 Woman’s Health Sciences Department, Gynecologic Section, Polytechnic University of Marche, 60121 Ancona, Italy; 13 Gynecologic and Obstetric Unit, Department of Medical, Surgical and Experimental Sciences, University of Sassari, 07100 Sassari, Italy; 14 Department of Obstetrics and Gynecology, University Sapienza of Roma, 00185 Rome, Italy; 15 Pediatric Gynecology Unit, Meyer Children’s Hospital IRCCS, 50139 Florence, Italy; 16 Department of Surgery, Fondazione IRCCS Istituto Nazionale dei Tumori di Milano, 20133 Milan, Italy

**Keywords:** epithelial ovarian cancer, clinical guidelines, gynecologic oncology, diagnosis, surgery, chemotherapy, recurrence, precision medicine, global health

## Abstract

Epithelial ovarian cancer (EOC) remains the most lethal gynecological malignancy worldwide, primarily due to late-stage diagnosis, limited early screening tools, and the aggressive nature of the disease. Increasing global efforts have been directed toward harmonizing clinical guidelines and ensuring equitable access to optimal care. This narrative review provides a comprehensive overview and critical comparison of the most recent international guidelines for ovarian cancer management, emphasizing similarities and discrepancies. Each section examines diagnostic, surgical, and therapeutic recommendations across all disease stages—from prevention to recurrence. Emerging advances that are reshaping the management of EOC such as preclinical models and the application of artificial intelligence are also discussed. This work provides an updated and practical synthesis to support clinicians, researchers, and policymakers in optimizing ovarian cancer management and guiding future research priorities in precision oncology.

## Simple Summary

This review provides an in-depth comparison of the most recent international guidelines for the management of epithelial ovarian cancer, examining how major organizations align or diverge in their diagnostic criteria, surgical strategies, and therapeutic recommendations across all disease stages. In addition, the review highlights emerging innovations—such as advanced preclinical models and the growing integration of artificial intelligence—that are reshaping clinical decision-making and may enhance personalized treatment pathways. Together, these elements offer clinicians and policymakers an updated framework to optimize patient outcomes.

## 1. Introduction

Epithelial ovarian cancer (EOC) is the deadliest form of gynecological cancer, with 314,000 women diagnosed and 207,000 deaths in 2020, based on the most recent estimates [1]. Management of ovarian cancer represents one of the main challenges in gynecologic oncology due to the combination of late diagnosis, limited early screening options, and the aggressive nature of the disease.

Currently, there is a strong international and global commitment toward the standardization of guidelines and equitable access to care for ovarian cancer, led at the forefront by the International Gynecological Cancer Society through the Global Equality Group in Ovarian Cancer Care (GEGOCC) project [2].

The objective of this work is to provide a practical and easily accessible overview and comparative analysis of the principal global guidelines regarding the management of patients with epithelial ovarian cancer. This study aims to highlight similarities and discrepancies, as well as current advancements and limitations in the treatment of epithelial ovarian cancer, serving as a valuable tool to support clinical decision-making and critical reflection in daily medical practice.

## 2. Materials and Methods

We conducted a systematic search of the latest published guidelines on treating epithelial ovarian cancer by reviewing websites of major international gynecological oncology societies across Europe, America, Asia, Africa, and Australia. Non-epithelial ovarian cancers were excluded because their extremely rare occurrence and unique pathogenesis make them a separate entity that requires individual analysis. According to the International Federation of Gynecology and Obstetrics (FIGO) report published in 2014 on the staging of ovarian cancer, this review includes ovarian, fallopian tube, and peritoneal cancer in the same category system.

The search strategy is updated to August 2025. The following guidelines were selected for review and comparison:

• National Comprehensive Cancer Network (NCCN) including NCCN Harmonized Guidelines for Sub-Saharan Africa and the Middle East and North Africa (MENA) [3]; American College of Obstetricians and Gynecologists (ACOG) guidelines will not be discussed.
• European Society of Medical Oncology (ESMO) [4,5,6].
• European Society of Gynaecological Oncology (ESGO) [7,8,9,10].
• Associazione Italiana Oncologia Medica (AIOM) [11].
• National Institute for Health and Care Excellence (NICE) [12,13,14].
• British Gynecological Cancer Society (BGCS) [15].
• Japan Society of Gynecologic Oncology (JSGO) [16].
• Australian Government Cancer Australia [17].

All guidelines were carefully reviewed and analyzed by five authors, and any differences in interpretation and comparison were resolved through discussions with other authors. The results are organized according to the diagnostic process, therapeutic interventions, follow-up protocols, and relapse management strategies.

## 3. Results

### 3.1. Screening and Prevention

Routine screening of the general population is currently not recommended by any international guidelines. At present, the proposed screening protocols lack strong evidence supporting their effectiveness in enabling early disease diagnosis in the general population and in reducing overall mortality [18]. Emerging and promising approaches include omics-based technologies related to liquid biopsy, such as the detection of circulating tumor DNA or microRNAs in bodily fluids [19,20].

Screening and early diagnosis are recommended within hereditary syndromes, given the evidence supporting the correlation between increased ovarian cancer risk and hereditary genetic mutations, primarily represented by BRCA1-2, homologous recombination gene panel germline mutations, and Lynch syndrome.

#### 3.1.1. Genetic Testing

Pre-test counseling should include an assessment of the patient’s understanding of genetic testing for cancer predisposition, covering its benefits, risks, and limitations, as well as the cancer risks linked to specific germline pathogenic variants (PV). Variants of uncertain significance (VUS) should not be used to alter medical management, as outlined by the NCCN guidelines. However, it is important to emphasize that these changes should be reevaluated periodically, as the international consortium ENIGMA regularly reviews them, which may lead to adjustments in the follow-up strategy.

According to NCCN and ESMO guidelines, genetic testing should not be offered to individuals under 18 years old (the age of legal adulthood in most countries) unless the results directly impact medical management. The main goals of genetic testing include enabling cascade testing in at-risk relatives, evaluating an individual’s risk for ovarian cancer, and guiding personalized risk management strategies.

Family history remains a fundamental component in estimating the probability of carrying a pathogenic mutation. Type of cancer, age at diagnosis, and ethnic background should always be investigated during the medical interview.

The criteria for accessing BRCA genetic testing differ across international guidelines, but they all emphasize family and personal cancer history. NICE, Australian, and NCCN guidelines include individuals with a personal or family history of ovarian cancer as eligible for testing. NICE recommends testing for those with a first-degree relative diagnosed with ovarian cancer and considers testing in second-degree relatives if testing the closer relative is not possible, especially in high-risk groups such as Ashkenazi, Sephardi Jewish, or Greenlandic populations. Likewise, the Australian guidelines focus on family clustering and suggest testing when there are two or more close blood relatives with breast or ovarian cancer on the same side of the family, particularly if other risk factors are present, such as early-onset cancer, bilateral breast cancer, male breast cancer, or Jewish ancestry.

The NCCN criteria are broader and more comprehensive, including individuals with any blood relative known to carry a pathogenic or likely pathogenic variant in a cancer susceptibility gene, those who meet testing criteria for related hereditary cancer syndromes (such as Li-Fraumeni, Cowden Syndrome, or Lynch syndrome), and individuals with a personal history of epithelial ovarian, fallopian tube, or peritoneal cancer at any age. NCCN also supports testing based on risk prediction models (i.e., CanRisk, which is endorsed by main oncological societies [21]), recommending it for individuals with a more than 5% likelihood of harboring a BRCA1-2 pathogenic variant, even if they do not meet classic family history criteria.

Regarding the ESMO criteria for genetic testing, the recommendations are fairly broad and mainly target individuals with a significant family history. This information is recommended to be integrated into risk prediction models to estimate the chance of carrying a hereditary pathogenic variant. Several European countries have developed their own, more detailed national guidelines. For example, AIOM offers detailed recommendations for all patients who are advised to undergo genetic counseling, based either on personal history or family history.

In the adapted NCCN guidelines for the MENA region, genetic testing is mainly recommended for individuals diagnosed with epithelial ovarian cancer or as cascade testing for first- and second-degree relatives. In contrast, genetic testing is not covered in the current Asian guidelines. A recent survey on genetic testing and counseling practices for women with breast and ovarian cancer in Asia [22] shows that BRCA1-2 testing and counseling are less commonly offered in Asian countries compared to Western regions. These differences may result from limited access to genetic testing services, a shortage of trained healthcare professionals, a lack of comprehensive training programs, and a low number of accredited laboratories across many parts of Asia.

#### 3.1.2. Surveillance Protocols in High-Risk Patients

If an individual at increased risk of ovarian cancer chooses to delay or decline risk-reducing surgery, they should be referred to appropriate surveillance protocols after thorough, informed counseling. It is important to clarify that, although surveillance may allow for earlier detection of ovarian cancer, it should not replace risk-reducing surgery, as it does not decrease the incidence of ovarian cancer and may produce false-positive or false-negative results. Table S1 details the surveillance protocols recommended by clinical guidelines; importantly, the NCCN does not endorse active surveillance protocols.

According to Australian guidelines, routine ovarian cancer screening with transvaginal ultrasound and serum CA125 is not recommended for women at high or potentially high risk. Instead, these women should be guided toward risk-reducing surgery or, if they are premenopausal and postponing surgery, toward chemo-preventive options such as oral contraceptives.

#### 3.1.3. Chemoprevention

Several studies in the literature have demonstrated the potential chemo-preventive effect of oral contraceptives in reducing ovarian cancer risk among BRCA1 and BRCA2 mutation carriers [23,24].

The use of oral contraceptives for chemoprevention varies among clinical guidelines. ESMO does not recommend them due to limited and conflicting evidence. NICE suggests considering their use only if the potential to reduce ovarian cancer risk outweighs the possible increase in breast cancer risk, and after discussing the timing of any planned risk-reducing surgery. Both NCCN and the Australian guidelines recognize combined contraception as a non-surgical option for risk reduction. In all cases, individuals should be fully informed of the potential risks and benefits before starting hormonal contraception for preventive purposes.

#### 3.1.4. Risk-Reducing Surgery

Bilateral salpingo-oophorectomy (BSO) is the standard risk-reducing intervention recommended for individuals carrying germline mutations in the BRCA1 or BRCA2 genes, as well as for those with other high-risk gene mutations or a diagnosis of Lynch syndrome. Table 1 summarizes current guideline-based indications for risk-reducing surgery. According to ESMO and NICE guidelines, risk-reducing salpingectomy—either as bilateral salpingectomy alone or followed by delayed oophorectomy—is not recommended outside the context of a clinical trial. In contrast, the NCCN guidelines recognize salpingectomy with delayed oophorectomy as a potential option for individuals with moderate-penetrance pathogenic variants, particularly in premenopausal patients who are not yet ready to undergo oophorectomy. This approach requires careful consideration of age-related cancer risk and family history. Several clinical trials investigating interval salpingectomy followed by delayed oophorectomy are currently underway (e.g., NCT02321228, NCT01907789, NCT04294927). The NCCN provides a detailed protocol for performing minimally invasive risk-reducing BSO, emphasizing thorough intra-abdominal evaluation (including the upper abdomen, bowel surfaces, appendix, and pelvic organs), collection of pelvic washings, and complete removal of the fallopian tubes and ovaries. This includes excising 2 cm of the proximal ovarian vascular pedicle or infundibulopelvic ligament, resection of the entire fallopian tube up to the uterine cornua, and removal of all peritoneal tissue surrounding the adnexa, with particular attention to areas of adhesion between the pelvic wall, ovary, and/or fallopian tube. Pathologists are encouraged to examine fallopian tubes according to the SEE-FIM (Sectioning and Extensively Examining the FIMbriated end) protocol to better identify tubal precursor lesions [25].

It is important to note that a residual risk of primary peritoneal carcinoma remains even after risk-reducing BSO in high-risk individuals. According to ESMO, there is no evidence to support continued gynecological screening following risk-reducing BSO. Literature also indicates that there is no conclusive evidence establishing a strong association between endometrial cancer and BRCA1-2 mutations, except for a slight increase in the risk of serous endometrial cancer in BRCA1-mutated carriers [26]. The updated ESGO guidelines on this topic [27] do not recommend routine hysterectomy at the time of BSO in BRCA1-2 carriers, emphasizing that this decision should be personalized based on each patient’s specific risk factors, including comorbidities, BMI, tamoxifen use, long-term hormone replacement therapy, and the presence of symptoms. Similarly, all international guidelines advise against routine risk-reducing hysterectomy in these patients.

Prophylactic BSO has significant physical and psychological effects. These include sudden menopause symptoms, emotional stress related to body image and fertility, and an overall effect on quality of life. Therefore, thorough pre- and post-surgery counseling is crucial to support informed choices and promote long-term mental and social well-being.

#### 3.1.5. Hormonal Replacement Therapy

Hormone replacement therapy (HRT) after risk-reducing BSO is a vital part of post-surgical care, especially for premenopausal women, due to the sudden onset of surgical menopause and its related symptoms. According to ESMO guidelines, systemic short-term HRT should be considered and discussed with eligible patients, while low-dose intravaginal estrogen options may help relieve genitourinary syndrome of menopause symptoms, particularly in those who are not candidates for systemic therapy. Both NICE and NCCN guidelines recommend offering HRT to women who have had BSO and no personal history of breast cancer, extending treatment until the typical age of natural menopause. The choice of HRT should depend on whether the woman has a uterus—combined estrogen-progestin therapy is recommended for women with a uterus to prevent endometrial hyperplasia, while estrogen-only therapy is suitable for women who have undergone a hysterectomy. Additionally, intrauterine progestin-releasing devices may be considered during surgery to help manage endometrial health protection.

The decision to start HRT should be made after a careful assessment of the patient’s symptom severity, personal and family history of cancer, and individual risk-benefit analysis.

#### 3.1.6. Fertility Preservation

Fertility preservation is a key consideration for individuals with BRCA1-2 mutations, especially those undergoing risk-reducing BSO at a young age. ESMO, NICE, and NCCN guidelines agree on offering comprehensive fertility counseling, including detailed discussions about oocyte and embryo cryopreservation, as well as the potential use of preimplantation genetic testing. Referring patients to fertility specialists is essential to ensure timely access to preservation options before surgical menopause or a natural decline in ovarian function reserve. Emerging evidence indicates that oocyte cryopreservation may be especially relevant for BRCA mutation carriers, who face a higher risk of accelerated ovarian aging and earlier menopause [28] compared to non-carriers. Several studies [29,30] have shown lower ovarian reserve markers, such as anti-Müllerian hormone levels, in BRCA1-2 mutated individuals, supporting the need for earlier and more proactive fertility preservation interventions.

Given the complex interaction between genetic risk, reproductive lifespan, and timing of risk-reducing surgery, fertility preservation should be incorporated into the broader multidisciplinary care plan.

### 3.2. Diagnosis

Preoperative diagnosis of epithelial ovarian carcinoma is a crucial step in managing patients, as it enables proper treatment planning, accurate staging, and better prognosis. Multiple scientific societies have created diagnostic guidelines that, while sharing core elements, vary in their approach, including the use of tumor markers, predictive ultrasound models, and advanced imaging recommendations.

#### 3.2.1. Clinical Examination

All international guidelines recommend a thorough medical history focused on suspicious symptoms such as abdominal distension, pelvic pain, early satiety, and urinary and intestinal changes, along with a complete physical examination of the abdomen, pelvis, and rectum (Table S2). According to ESGO and ESMO recommendations, the physical examination should include abdominal, pelvic, and rectal assessments, evaluation of extrapelvic lymph nodes (inguinal, supraclavicular, and axillary), and inspection of the chest. The BGCS provides similar guidance, emphasizing the relevance of patient age, personal and family cancer history, and the presence of ascites or fixed masses as potential indicators of malignancy. AIOM does not specify the preoperative diagnostic phase, assuming an appropriate initial assessment before surgical staging. African-adapted NCCN guidelines adopt a clinically essential yet thorough approach, highlighting the importance of a complete history even when advanced resources are unavailable. They recommend bimanual pelvic examination, abdominal assessment for ascites or distension, and a detailed family and nutritional history.

#### 3.2.2. Ultrasound and Predictive Models

Pelvic ultrasound is regarded as a crucial first-line diagnostic tool for assessing adnexal masses in all guidelines (Table S3). Specifically, transvaginal ultrasound is universally advised for the initial assessment of suspicious adnexal masses. Evidence-based statements on the ultrasonographic diagnosis of ovarian tumors and predictive models have been published in 2021 after a consensus approved by a multidisciplinary international group [31]. The diagnostic accuracy of ultrasonography in differentiating benign from malignant adnexal masses largely depends on operator expertise. The European Federation of Societies for Ultrasound in Medicine and Biology defines three levels of gynecological ultrasound competence, combining theoretical and practical training. Level III (expert) sonographers—those primarily dedicated to clinical practice, teaching, or research—demonstrate superior diagnostic performance [32]. To promote standardization, the International Ovarian Tumor Analysis (IOTA) group developed predictive ultrasound models—Simple Rules, LR1, LR2, and ADNEX—which outperform traditional indices such as the Risk of Malignancy Index (RMI). The ADNEX model not only estimates malignancy risk but also classifies adnexal lesions as benign, borderline, stage I malignant, stage II–IV malignant, or metastatic. These models improve diagnostic accuracy even among less experienced operators. Expert sonographers can further refine diagnosis through detailed morphological assessment, often recognizing characteristic patterns suggestive of specific histological subtypes (e.g., serous, mucinous, endometrioid).

NICE and BGCS guidelines specify that when symptoms indicate ovarian cancer, CA-125 serum levels along with both transabdominal and transvaginal pelvic ultrasound should be performed without delay. A CA-125 level of 35 U/mL or higher along with a suspicious ultrasound finding, warrants urgent referral to a gynecologic oncology specialist. African guidelines highlight the importance of both transabdominal and transvaginal ultrasound as primary diagnostic tools, even in low-resource settings. While IOTA models are not officially adopted, NCCN-adapted guidelines for Sub-Saharan Africa acknowledge the predictive value of structured ultrasound criteria and encourage the systematic use of ultrasound in limited-resource environments.

#### 3.2.3. Second Level Imaging

Advanced radiological investigations are essential for preoperative staging, assessing resectability, and detecting distant metastases. ESGO, BGCS, ESMO, and JSGO recommend contrast-enhanced chest-abdomen-pelvis computed tomography (CT) as the preferred imaging modality (Table S4). CT is used for preoperative staging and evaluating the extent of disease. However, it has limitations in detecting low-volume peritoneal carcinomatosis and mesenteric infiltration. According to ESGO, Magnetic Resonance Imaging (MRI) should not be routinely employed but may serve as a second-level option for young women with indeterminate masses or suspected non-epithelial neoplasms, especially when fertility preservation is a consideration. Positron Emission Tomography (PET)-CT is not recommended as a routine test but can be helpful in assessing thoracic lymphadenopathy or extraperitoneal lesions that are not characterized by CT or MRI. NCCN-adapted guidelines for Sub-Saharan Africa reserve advanced imaging for indeterminate lesions or cases where it could influence the treatment plan.

#### 3.2.4. Tumor Markers

Recommendations are summarized in Table S5. All guidelines recognize CA-125 as the primary serum marker but acknowledge its limitations, including low sensitivity in early stages and potential elevation in benign conditions such as endometriosis, fibroids, and inflammatory diseases. ESGO-ESMO recommends combining CEA and CA19-9 to identify possible gastrointestinal tumors, especially in mucinous masses. In young patients, BGCS and ESGO recommend an extended panel including AFP, β-hCG, LDH, Inhibin B, and AMH to detect germ cell or granulosa cell tumors. According to NCCN guidelines, additional serum markers should be considered based on the suspected histologic subtype. NCCN-adapted guidelines for Sub-Saharan Africa guidelines incorporate this approach while emphasizing serum markers such as AFP, β-hCG, LDH, Inhibin B, and AMH—especially for younger women or suspected non-epithelial tumors. They also recognize markers less commonly used in Western contexts (such as FSH, transferrin, and HE4), though routine use is not advised.

According to ESGO guidelines, HE4 may be used as a complementary marker to CA-125 in selected clinical scenarios, particularly in younger patients or when mucinous, non-epithelial, or extra-adnexal tumors are suspected. It can aid in differentiating benign from malignant ovarian masses when CA-125 levels are nonspecifically elevated, such as in endometriosis or inflammatory conditions. Conversely, ESMO, BGCS, and NCCN recommendations indicate that HE4 does not provide superior diagnostic accuracy compared to CA-125 alone. Consequently, routine use of HE4 in the standard preoperative evaluation is not advised. HE4 may hold potential as a prognostic or follow-up marker, but its role remains non-standardized.

#### 3.2.5. Histological and Cytological Diagnosis

The need for tissue confirmation depends on the planned treatment strategy. Both BGCS and ESGO recommend histological or cytological diagnosis before initiating neoadjuvant chemotherapy, except in exceptional circumstances. Primary surgery is acceptable when malignancy is highly suspected, even without prior biopsy. Ascitic or pleural fluid analysis is included in FIGO staging; however, cytology alone is not considered sufficient for definitive diagnosis due to possible false results, particularly in cases of reactive mesothelium or borderline tumors. Cytology is acceptable only when biopsy is contraindicated, such as in patients with poor performance status. According to NCCN guidelines, diagnosis should ideally be established by tissue biopsy supported by cytology. When immediate surgery is not feasible, ultrasound- or CT-guided percutaneous biopsy is recommended, while definitive diagnosis in surgical candidates is based on intraoperative and histopathological findings.

### 3.3. Pre-Operative Work-Up

Table S6 summarizes the comparison data of current guidelines.

#### 3.3.1. Pre-Operative Imaging

Major international societies agree on the importance of high-quality imaging and targeted laboratory assessments for the initial evaluation and surgical planning in patients with suspected ovarian cancer. However, some differences exist in their specific recommendations and clinical priorities. ESMO-ESGO, NCCN, and BGCS guidelines all recommend using contrast-enhanced CT of the chest, abdomen, and pelvis as the first choice for imaging to determine disease extent and resectability. Expert-performed transvaginal ultrasound is also considered useful for evaluating local tumor spread and resectability, especially within the pelvis and abdominal cavity. It is crucial to distinguish pre-operatively between local tumor extension—such as from the omentum to the capsule of the spleen or liver (stage IIIC)—and parenchymal metastases to these organs (stage IVB), as this distinction has important prognostic and treatment implications.

#### 3.3.2. Laparoscopic Evaluation Prior to Resection—Assessment Laparoscopy

Current clinical practice guidelines agree in recommending an initial laparoscopic assessment for newly diagnosed ovarian cancer patients with suspected advanced-stage disease. The main goal is to determine the extent of the disease and evaluate the feasibility of optimal cytoreduction, recognizing that laparoscopy might underestimate disease presence in areas such as the subdiaphragmatic peritoneum, porta hepatis, and deep retroperitoneal nodes. Currently, there is no universally accepted scoring system. Among existing laparoscopic predictive models for potential primary cytoreduction, the Fagotti Predictive Index Value [33] and Peritoneal Cancer Index (PCI) [34] are the most widely used. Although the PCI was initially validated in colorectal carcinomatosis and peritoneal mesothelioma, growing evidence [35,36] supports its use in advanced epithelial ovarian cancer. However, a standardized, universally accepted PCI cut-off specific to ovarian cancer has yet to be widely validated.

Deciding between primary cytoreductive surgery and neoadjuvant chemotherapy is a complex process that involves considering patient performance status, tumor biology, imaging results, and institutional surgical expertise. Developing and validating reliable preoperative tools to identify patients who are unlikely to benefit from primary debulking can help reduce surgical complications and support personalized treatment plans. In this context, surgical complexity scoring systems are important tools for preoperative evaluation and intraoperative decision-making in patients undergoing cytoreductive surgery for advanced ovarian cancer. The Aletti Surgical Complexity Score [37] measures surgical burden by assigning weighted points to specific procedures (e.g., bowel resections, peritonectomies, diaphragmatic stripping), producing an overall score that is categorized as low, intermediate, or high complexity. Higher surgical complexity has been linked to increased risk of perioperative complications, especially in older patients or those with comorbidities, as indicated by performance status or American Society of Anesthesiologists (ASA) grade. The Vizzielli score [38], meanwhile, is a predictive model designed specifically to evaluate the risk of postoperative morbidity and mortality by integrating disease extent and surgical complexity with patient-related factors. This scoring system considers variables such as the Fagotti Predictive Index Value, ascites, CA125 levels, and the patient’s ECOG performance status score. These scoring tools aid clinical decision-making by balancing surgical intensity with patient safety.

#### 3.3.3. Perioperative Management and Patient Optimization

International guidelines increasingly emphasize structured perioperative management and patient optimization to improve outcomes in advanced ovarian cancer surgery. The ESGO guidelines [8] provide a comprehensive framework for perioperative care aimed at reducing surgical morbidity and mortality through a standardized approach that includes frailty assessment, prehabilitation, and Enhanced Recovery After Surgery (ERAS) protocols [39,40]. ERAS is a multimodal strategy designed to reduce surgical stress and promote faster recovery through preoperative education, nutritional optimization, intraoperative normothermia and fluid balance, and postoperative early feeding, mobilization, and opioid-sparing analgesia. Similarly, BGCS highlights the importance of patient optimization via frailty screening, personalized prehabilitation, and ERAS implementation. Both ESGO and BGCS emphasize that frailty, rather than chronological age, should guide surgical decisions.

The NCCN emphasizes assessing nutritional status before surgery, especially in older patients, due to its influence on surgical complications, cytoreductive success, and survival. The ESMO-ESGO consensus discourages excluding treatments (including diagnostic procedures, clinical trials, and specific therapies) solely based on age. It recommends a comprehensive geriatric assessment for patients over 70 years or those with two or more comorbidities, covering functional, nutritional, and psychological aspects.

In summary, all guidelines emphasize a personalized, multidisciplinary perioperative approach, with ERAS protocols, frailty and nutritional assessments, and prehabilitation as key elements to improve the tolerance of any medical and surgical interventions and to reduce complications associated with complex gynecologic oncology surgery.

#### 3.3.4. Molecular Testing

Molecular testing has become essential for personalized management of ovarian cancer, allowing for more accurate diagnosis, prognosis, and treatment planning [41].

ESMO recommends that all patients with high-grade ovarian cancer undergo germline and/or somatic BRCA1/2 mutation testing at diagnosis. Additionally, homologous recombination deficiency (HRD) testing is advised in advanced high-grade tumors to guide treatment decisions. The ESMO–ESGO consensus further emphasizes that BRCA testing should be performed on treatment-naïve tumor samples whenever possible. This recommendation is specifically for patients with high-grade non-mucinous tubo-ovarian carcinomas, regardless of disease stage. While routine testing for non-BRCA homologous recombination gene mutations is currently limited to research settings, genomic instability assays are advised for patients with BRCA wild-type stage III–IV tubo-ovarian carcinomas to guide first-line maintenance therapy decisions. The ESMO–ESGO consensus also emphasizes the importance of mismatch repair (MMR) immunohistochemistry (IHC) and/or microsatellite instability (MSI) testing in ovarian endometrioid and clear cell carcinomas to identify cases linked to Lynch syndrome. Likewise, the NCCN guidelines endorse comprehensive genetic risk assessment, recommending both germline and somatic testing—including BRCA1-2 mutation status and HRD assessment—at diagnosis in patients with advanced ovarian cancer to determine eligibility for Poly (ADP-ribose) polymerase (PARP) inhibitor maintenance therapy. This approach also applies in the recurrence setting to identify patients who might benefit from targeted therapies. The NICE guidelines support panel-based germline genetic testing for all adults with non-mucinous high-grade epithelial ovarian cancer, recognizing it as a quality indicator. These recommendations are supported by the BGCS, which also highlights that, given the estimated 12–14% prevalence of Lynch syndrome in clear cell ovarian cancers, universal MMR IHC testing should be conducted in this histological subtype. The same advice applies to endometrioid ovarian carcinomas. The rationale involves both detecting Lynch syndrome and the potential importance of immune checkpoint inhibitors in relapsed or advanced MMR-deficient disease. The AIOM guidelines suggest that all patients with ovarian cancer—regardless of whether the tumor is low-grade or high-grade, as long as it is non-mucinous and non-borderline histology—should undergo BRCA mutation testing at diagnosis, adopting a broader approach.

### 3.4. Primary Cytoreductive Surgery: Upfront and Interval Debulking

Debulking surgery is the cornerstone of ovarian cancer treatment. The main goal of debulking surgery is to achieve maximum cytoreduction, where optimal cytoreduction is defined as residual disease less than 1 cm in size or thickness, as data in the literature show better survival outcomes when there is no residual tumor after surgery [42].

Comparison of data is outlined in Table 2.

#### 3.4.1. Serous Tubal Intraepithelial Cancer (STIC)

The reported frequency of serous tubal intraepithelial carcinoma (STIC) in risk-reducing BSO among high-risk populations—specifically carriers of BRCA mutations—varies widely from 0.4% to 8.5%. However, this rate is roughly ten times higher than that seen in low-risk groups. The presence of STIC has been linked to a greater risk of developing primary peritoneal carcinoma later on, emphasizing the need for thorough peritoneal assessment during surgery.

Accordingly, the ESMO–ESGO consensus conference recommends peritoneal staging when STIC is identified. Newly presented ESGO recommendations at the 2025 ESGO congress [27] clarify the role of peritoneal staging in cases of isolated STIC. When isolated STIC is found during RRBSO, surgical staging should be considered only if peritoneal cytology—performed during risk-reducing surgery—is positive. Conversely, if an isolated STIC is discovered after salpingectomy for benign reasons, removing the ovaries, performing peritoneal cytology, thoroughly inspecting the peritoneum, and biopsying any suspicious lesions are advised. Additionally, hysterectomy should be considered. If a hysterectomy is not performed, endometrial sampling is recommended. Lymphadenectomy is not mandatory, and adjuvant chemotherapy is not advised for surgically staged, isolated STIC. In contrast, the NCCN guidelines offer two main management options for isolated STIC without evidence of invasive carcinoma: observation alone, with or without serial CA-125 monitoring; or surgical staging, followed by either observation or chemotherapy if invasive disease is found. These recommendations adopt a risk-based approach that incorporates clinical judgment based on intraoperative and histopathological findings.

BGCS guidelines further refine the management pathway. In cases where isolated STIC is diagnosed after early bilateral salpingectomy alone, and both peritoneal cytology and imaging are normal, performing a complete bilateral oophorectomy is highly recommended to decrease the risk of progression. If peritoneal cytology is positive, then comprehensive surgical staging is necessary to rule out or identify any hidden invasive disease.

#### 3.4.2. Borderline Ovarian Tumor (BOT)

Borderline ovarian tumors (BOTs) represent a rare entity, accounting for 10 to 20% of all ovarian epithelial neoplasms. They exhibit good prognosis with an overall survival rate higher than 90% in 10 years and have a recurrence rate between 5% and 34%; approximately 70% of BOTs recur with similar borderline histological features, most commonly involving the contralateral adnexa. Malignant transformation occurs in about 30% of cases, especially when invasive peritoneal implants are detected at first diagnosis, in the form of low-grade carcinomas [43]. Serous BOTs (sBOTs) are the most common subtype, being associated with peritoneal implants in one-third of cases [44]. Prognosis is significantly poorer in patients with invasive growth pattern implants. Due to the complexity of histopathological differentiation between invasive and non-invasive implants, expert pathological review is recommended to ensure accurate diagnosis and prognostic stratification.

According to the ESMO–ESGO consensus conference, peritoneal and omental disease should be surgically removed to enable a definitive histological assessment of the implant type, while noting that such implants are specific to serous subtypes and should not be interpreted similarly in mucinous borderline tumors (mBOTs), where they are usually metastases from either primary mucinous ovarian carcinoma or extra-ovarian gastrointestinal tumors [45].

Regarding fertility preservation, fertility-sparing surgery—defined as preserving the uterus and at least part of one ovary—is the standard care for women of reproductive age with BOTs. Unilateral salpingo-oophorectomy is preferred over cystectomy to minimize the risk of invasive recurrence. In contrast, for postmenopausal women, BSO with or without hysterectomy is the standard surgical approach. Routine appendectomy is not necessary, even in the mucinous subtype, if the appendix appears macroscopically normal during surgery. Likewise, there is no evidence to support routine systematic lymphadenectomy.

The role of hysterectomy remains a subject of ongoing debate. Notably, the ESMO–ESGO guidelines do not explicitly address the role of hysterectomy. A large national, multicenter, retrospective cohort study involving postmenopausal patients with BOT from 20 Italian centers [46] found that hysterectomy was linked to a significantly lower risk of recurrence. However, it did not affect overall survival or disease-specific mortality. These findings suggest that hysterectomy could be incorporated into the surgical management of BOTs in postmenopausal women to reduce recurrence risk. A recent meta-analysis [47] supports these observations: uterine-sparing surgery is associated with an increased risk of recurrence but does not influence overall or disease-specific survival. Furthermore, considering the generally indolent nature of BOT recurrences—typically managed successfully with repeat surgery and with <5% risk of invasive transformation—uterine preservation might be considered even in women not seeking future fertility, especially in cases of incidental BOT diagnosis where hysterectomy was not performed during initial surgery.

The BGCS guidelines emphasize fertility preservation in younger women, recommending a conservative approach when possible. Routine hysterectomy is not advised unless the uterus is macroscopically involved and the patient does not want fertility. Appendectomy should only be performed if the appendix appears abnormal. In early-stage sBOTs, bilateral cystectomy may be suitable, and ipsilateral adnexectomy is not routinely advised unless high-risk features, such as micropapillary patterns, are present. Restaging surgery—including peritoneal cytology, peritoneal biopsies, omentectomy, and, when indicated, ipsilateral oophorectomy—is recommended for serous BOTs with micropapillary features or if intraoperative assessment during initial surgery was inadequate. For early-stage mBOTs, unilateral adnexectomy is suggested if cystectomy was initially performed. Restaging is also recommended if the appendix was not assessed during primary surgery. In postmenopausal women with suspected unilateral or bilateral BOTs, BSO is advised before surgery. Lymphadenectomy is generally not recommended, even in advanced stages, unless enlarged lymph nodes are visible. The main surgical aim in advanced or recurrent BOTs remains the complete removal of the visible tumor.

In practice, clinical management varies significantly between countries. For example, AIOM guidelines similarly recommend conservative surgery with complete staging in premenopausal women. This includes omentectomy, peritoneal washing with cytology, removal of any visible peritoneal lesions, and multiple peritoneal biopsies. Pelvic and para-aortic lymphadenectomies are not routinely advised. In mucinous tumors, appendectomy should be performed as part of the staging process. The Collège National des Gynécologues et Obstétriciens Français [48] advises against routine hysterectomy in early-stage serous or mucinous BOTs but recommends it in endometrioid BOTs when fertility preservation is not desired.

The Japanese guidelines adopt a more comprehensive approach, recommending BSO, total hysterectomy, omentectomy, peritoneal cytology, and multiple abdominal biopsies as the standard procedure. The NCCN guidelines specifically focus on determining the presence or absence of invasive implants, which influences the need for postoperative therapy. Their standard surgical method includes total hysterectomy, BSO, and debulking of visible disease, while lymphadenectomy and omentectomy are not required.

In conclusion, differences mainly occur in how postmenopausal women are managed, especially regarding the usefulness of hysterectomy and the decision to perform appendectomy in mucinous subtypes. These differences stem from varying interpretations of the evidence and also reflect different cultural and clinical priorities, such as reproductive preservation, surgical aggressiveness, and tolerance for recurrence risk. To align guidelines, more prospective data and long-term follow-up studies are needed, particularly concerning the prognostic importance of hysterectomy and how incidental BOT diagnoses are managed.

#### 3.4.3. Low Grade Serous Ovarian Cancer (LGSC)

Low-grade serous carcinoma (LGSC) presents unique therapeutic challenges because of its resistance to chemotherapy. Surgical management is crucial in the treatment approach, and international guidelines offer recommendations tailored to these biological features.

The ESMO–ESGO consensus conference recommends removing all visible peritoneal implants along with formal peritoneal staging as the standard approach. Routine systematic lymphadenectomy is not advised; instead, resection should be limited to enlarged or suspicious lymph nodes. Regarding surgical strategy, primary debulking surgery (PDS) is preferred when complete resection seems feasible, considering the limited chemosensitivity of LGSC. For patients with low chances of optimal cytoreduction, poor performance status, or histological subtypes that respond better to chemotherapy, neoadjuvant chemotherapy (NACT) followed by interval debulking surgery (IDS) may be an option. Likewise, the NCCN guidelines recommend comprehensive surgical staging as the key initial management step. These guidelines explicitly discourage routine use of NACT in LGSC due to its poor chemotherapy response, reinforcing the preference for upfront surgery when possible. The BGCS guidelines align closely with these principles, emphasizing that PDS aimed at achieving no visible residual disease remains the best treatment approach. The focus on complete cytoreduction highlights both the chemo-resistant nature of LGSC and the importance of residual disease in predicting long-term outcomes.

In summary, according to ESMO–ESGO, NCCN, and BGCS guidelines, there is widespread agreement that aggressive surgical cytoreduction should be prioritized in managing LGSC, with the exception that the ESMO-ESGO guidelines are the only ones to include a specific recommendation against routine systematic lymphadenectomy. All three organizations emphasize the limited role of chemotherapy in this context and highlight the importance of complete macroscopic resection to improve prognosis. The use of NACT is limited and considered appropriate only under specific clinical conditions, such as low resectability or poor performance status. These unified recommendations reinforce the idea that surgery remains the primary approach for effective treatment in LGSC.

#### 3.4.4. High Grade Serous Ovarian Cancer

**Early stages (I–II):**

Across major international guidelines, comprehensive surgical staging remains the standard of care for presumed early-stage (FIGO I–II) ovarian cancer. Total hysterectomy, BSO, infracolic omentectomy, and peritoneal cytology (including either ascitic fluid or peritoneal washing before tumor manipulation) are consistently recommended. Inspection and palpation of the entire abdominal cavity, along with peritoneal biopsies—either targeted (from visible lesions) or random (when no disease is apparent)—are broadly endorsed. Most guidelines (ESMO, ESGO, NCCN, JSGO) recommend pelvic and para-aortic lymphadenectomy, usually extending to the level of the left renal vein. BGCS guidelines take a more nuanced approach by suggesting pelvic and para-aortic lymphadenectomy in the absence of peritoneal spread, either for prognostic assessment or to guide adjuvant treatment decisions. Japanese guidelines offer the most detailed site-specific biopsy recommendations, including the pouch of Douglas, abdominal wall, diaphragm, bowel, and mesentery. NCCN uniquely mentions the option of PAP scrapings from the diaphragmatic surface when biopsies are not feasible.

**Restaging of incidentally diagnosed ovarian cancer:**

International guidelines present varying approaches to managing incidentally diagnosed ovarian cancer, especially concerning the need for restaging procedures.

The NCCN recommends that in patients with epithelial ovarian cancer diagnosed after a prior surgical procedure, further surgical staging is unnecessary if no residual disease is evident and adjuvant chemotherapy is planned. For selected histologic subtypes with presumed stage IA-IB disease, observation may be considered. In such cases, surgical staging can help identify patients who are eligible for observation or reduced chemotherapy. If staging confirms stage IA–IB, observation is appropriate; if higher-stage disease is detected, adjuvant chemotherapy is advised. Surgical staging may also guide decisions regarding eligibility for maintenance therapy in presumed stage IA–IC patients without residual disease. The ESGO recommends a second surgical procedure when early-stage carcinoma is incidentally identified during surgery for presumed benign disease, provided comprehensive staging was not initially performed. However, appendectomy is not mandatory in mucinous histology if the appendix has been assessed and found to be normal. Minimally invasive surgical staging is considered acceptable. Conversely, the Japanese guidelines recommend staging with a laparotomic approach in such cases. If advanced-stage ovarian cancer is diagnosed incidentally, debulking surgery following chemotherapy is advised. The BGCS supports considering pelvic and para-aortic lymphadenectomy as a secondary staging procedure after confirming malignancy. This may be performed for prognostic purposes or when nodal status is expected to inform adjuvant treatment decisions meaningfully.

**Advanced stages (III–IV):**

The standard of care for initial management of advanced tubo-ovarian carcinoma involves either PDS or, in patients deemed unsuitable for upfront surgery, NACT followed by IDS. PDS remains the preferred approach when complete macroscopic resection is considered achievable with acceptable morbidity, as achieving residual disease < 1 cm has consistently been linked to significantly improved progression-free survival (PFS) and overall survival (OS).

To date, four randomized clinical trials [49,50,51,52], alongside the recently published TRUST trial [53], have directly compared PDS and IDS. Available data suggest that IDS is associated with higher complete cytoreduction rates and lower perioperative morbidity and mortality showing no significant difference in OS between these surgical approaches. However, interpretation must be approached with caution due to considerable heterogeneity across studies in terms of patient populations, chemotherapy regimens, and surgical protocols. NACT appears to be an accepted alternative in patients unlikely to achieve complete cytoreduction at diagnosis and in poor surgical candidates due to age, comorbidities, frailty, or poor performance status. Appropriate patient selection for PDS or NACT must be undertaken in a certified ovarian cancer center, in accordance with the ESGO quality indicators for ovarian cancer surgery [54], and should be guided by a multidisciplinary team approach. In line with this, the NICE guidelines have also introduced and recently updated a set of quality indicators aimed at standardizing and improving the care pathway for patients with ovarian cancer [55].

Across major international guidelines, maximal cytoreductive surgery—including extensive procedures such as diaphragmatic stripping, peritonectomies, bowel and liver resections, splenectomy, partial gastrectomy, pancreatic tail resection, and removal of extra-abdominal disease—with the goal of no visible residual disease is considered the gold standard for advanced epithelial ovarian cancer. In this context, multidisciplinary evaluation is recommended to assess resectability and determine optimal treatment sequencing. ESGO provides detailed criteria against abdominal debulking, such as diffuse deep infiltration of the small bowel mesentery, extensive small bowel involvement risking short bowel syndrome, and infiltration of critical structures (e.g., pancreas head, celiac trunk). Non-resectable metastases include central or multi-segmental liver lesions, multiple parenchymal lung or brain metastases, and unresectable nodal disease. The ESMO-ESGO consensus emphasizes maximal surgical effort and recommends resection of isolated parenchymal liver metastases to achieve complete cytoreduction.

Surgery should be performed by experienced gynecologic oncologists at high-volume, specialized centers with access to multidisciplinary teams, as the surgeon’s expertise significantly influences the likelihood of achieving complete cytoreduction, improved survival outcomes, and low postoperative morbidity. NCCN guidelines tailored for sub-Saharan Africa specify that, in the absence of a gynecologic oncologist, an experienced gynecologist can perform the surgery with appropriate consultation.

**Surgical approach: laparoscopy versus laparotomy:**

Current international guidelines generally do not recommend laparoscopy for debulking procedures in ovarian cancer. Instead, a vertical midline laparotomy is advised for surgical staging, PDS, and IDS. The NCCN guidelines permit minimally invasive surgery (MIS) only in highly selected early-stage cases or, in exceptional circumstances, for IDS in patients where optimal cytoreduction is achievable and an experienced surgeon performs the procedure. Similarly, ESGO recommends midline laparotomy as standard but allows MIS for stage I–II disease when performed by a gynecologic oncologist, with strict precautions to prevent intraoperative tumor rupture. This preference for open surgery is primarily based on the need for accurate macroscopic evaluation of the abdominal cavity and to reduce the risk of tumor rupture during resection, which could compromise staging accuracy and oncologic outcomes. The literature shows promising data, as several studies indicate that MIS does not negatively impact survival or surgical morbidity [56]; we are currently awaiting the results of the LANCE trial [57]—a randomized controlled trial comparing MIS to open surgery in patients diagnosed with EOC who responded to NACT. No widely accepted scoring system currently exists to assess MIS feasibility in this setting; some authors have proposed predictive models [58] but further research is needed.

**Role of lymphadenectomy:**

The LION study [59] demonstrated that systematic pelvic and para-aortic lymphadenectomy offers no survival benefit in patients with advanced ovarian cancer (IIB–IVB) who have undergone complete macroscopic intra-abdominal resection and present with clinically and radiologically normal lymph nodes. These findings support the idea that microscopic nodal metastases in advanced disease should be managed with systemic chemotherapy rather than surgical removal. As a result, the primary role of systematic lymphadenectomy may shift toward staging rather than providing a therapeutic benefit. In early-stage disease, improved diagnostic imaging and sentinel lymph node (SLN) biopsy may help identify patients who could safely avoid full lymphadenectomy. The recent SELLY study [60]—a multicenter, prospective, phase II trial conducted from 2018 to 2022—failed to demonstrate enough sensitivity compared to standard systematic lymphadenectomy to support routine SLN biopsy in this setting. Several studies [61] have explored the potential role of SLN in ovarian cancer and have reported SLN sensitivity ranging from 73.3% to 100%, and accuracy between 96% and 100%, especially when ultrastaging was used. Given the ongoing debate about the therapeutic value of systematic lymphadenectomy—particularly in early-stage disease—additional research into SLN biopsy is needed. This approach may reduce surgical morbidity while still ensuring oncologic safety.

In early-stage ovarian cancer, the ongoing LOVE trial (NCT04710797), a multicenter, randomized phase III study, aims to evaluate the efficacy and safety of comprehensive staging with and without lymphadenectomy in FIGO stage IA–IIB epithelial ovarian and fallopian tube carcinomas. Its results are expected to provide high-level evidence to guide future clinical decisions. The rationale for performing lymphadenectomy in early-stage disease lies in its primarily diagnostic role rather than its therapeutic role. If micrometastases are found, the disease is reclassified as stage III, which alters subsequent management—including the potential use of PARP inhibitors. A potential limitation of the LOVE trial is that the risk of omitting lymphadenectomy may lead to missing patients who are actually stage III, thereby denying them access to PARP inhibitors. Evidence suggests that OS improves only when PARP inhibitors are used as part of first-line maintenance therapy [62,63].

Regarding lymphadenectomy at the time of interval debulking surgery, guidelines do not clarify its role and do not distinguish between primary and interval debulking surgery. Starting from a study by Fagotti et al. [64], the current existing literature supports the secondary role of lymphadenectomy during IDS—unless pre-operative suspicion or incidental lymph node findings—as there is no evidence of significant advantage in terms of OS and PFS while being associated with higher post-operative complications [65].

International guidelines offer recommendations for pelvic and para-aortic lymphadenectomy in epithelial ovarian cancer, with a general consensus emerging on the selective use of lymph node dissection based on disease stage and nodal status assessed through imaging and intraoperative evaluation. Most guidelines agree that systematic pelvic and para-aortic lymphadenectomy should not be performed in advanced-stage ovarian cancer when lymph nodes are clinically and radiologically negative, due to the lack of survival benefit and increased morbidity. It is important to note that, in this context, there is some inconsistency and disagreement among the guidelines regarding the definition of advanced-stage disease. Specifically, the NCCN and Japanese guidelines define advanced stages as those beyond stage IIB, while the ESMO-ESGO guidelines consider only stages III–IV, and the BGCS and NICE guidelines include stages II–IV as advanced. Regarding early stages, Japanese, ESMO, and NCCN support systematic lymphadenectomy for staging purposes. According to NICE guidelines, systematic retroperitoneal lymphadenectomy (including both para-aortic and pelvic nodes) is recommended only for patients with disease presumed to be confined to the ovaries. BGCS guidelines permit pelvic and para-aortic lymphadenectomy when there is no peritoneal spread, especially when nodal status is expected to influence adjuvant treatment decisions. Frozen section analysis is advised before lymphadenectomy to confirm malignancy.

**Role of neoadjuvant chemotherapy:**

According to the ESMO guidelines, an initial treatment involves three cycles of neoadjuvant paclitaxel–carboplatin, either alone or combined with bevacizumab. If interval debulking surgery is not possible after these initial cycles, an additional three cycles of chemotherapy are recommended. The use of hyperthermic intraperitoneal chemotherapy (HIPEC) during IDS is not currently standard practice. However, growing evidence in the literature indicates potential benefits. Notably, the OVHIPEC-1 trial [66] showed improved PFS (10.7 months versus 14.3 months in the IDS plus HIPEC group) and OS (33.3 months versus 44.9 months in the IDS plus HIPEC group) at 10-year follow-up, supporting earlier findings by van Driel et al. [67]. These results, while encouraging, require further validation, especially through subgroup analyses that consider surgical prognostic factors such as residual disease and the rate of optimal cytoreduction.

The NCCN guidelines offer a more stratified approach based on treatment response. For patients who show a favorable response to NACT, IDS followed by consideration of HIPEC is recommended. In cases of stable disease, IDS with or without HIPEC may be options, or systemic therapy may be continued to complete six cycles, aligning with treatment strategies for persistent or recurrent disease. HIPEC may be considered during IDS in stage III patients and in stage IV patients who have responded well to NACT, including those with complete resolution of stage IV findings (such as pleural effusion), or in whom the metastatic disease is deemed resectable.

ESGO also endorses the use of NACT, with a flexible approach to delayed IDS in patients who do not undergo surgery after the initial three cycles. Delayed debulking beyond three cycles may be considered on a case-by-case basis.

The BGCS guidelines recommend using NACT followed by IDS after three cycles of platinum-based chemotherapy for patients unlikely to achieve no residual disease through primary debulking surgery or who are medically unfit for extensive surgery. HIPEC may be considered during IDS for newly diagnosed ovarian cancer patients, in accordance with NICE interventional procedure guidance, but it should only be offered in highly specialized centers.

BGCS highlights the increasing importance of the Chemotherapy Response Score (CRS) [64], a histopathological grading system based on evaluating the omental resection specimen obtained during interval debulking surgery. CRS provides a standardized way to assess tumor response to NACT, classifying patients into three groups: complete or near-complete response (CRS 3), partial response (CRS 2), and minimal or no response (CRS 1). The International Collaboration on Cancer Reporting endorses CRS as a valuable prognostic biomarker, especially useful in guiding clinical decisions when a suboptimal response to NACT is observed. Although CRS has prognostic value, current evidence does not support changing standard chemotherapy protocols solely based on CRS categorization. Nonetheless, CRS remains an active area of research to evaluate its predictive power and explore its potential role in customizing post-operative treatment strategies.

#### 3.4.5. Special Subtypes (Mucinous, Endometrioid, Clear Cell Ovarian Cancer)

The ESMO–ESGO consensus conference recommends that comprehensive surgical staging should be the standard of care for early-stage high-grade endometrioid carcinoma, clear cell carcinoma, and high-risk mucinous ovarian carcinoma. The procedure should include total abdominal hysterectomy, bilateral salpingo-oophorectomy, omentectomy, systematic pelvic and para-aortic lymphadenectomy, peritoneal biopsies, and peritoneal washings for cytological analysis evaluation. In cases of advanced-stage mucinous carcinoma, PDS is usually preferred over IDS due to the lower chemosensitivity of these tumors. Notably, reoperation or re-evaluation solely for the purpose of performing an appendectomy is not considered necessary when the appendix has already been examined intraoperatively and found to be macroscopically normal, even in the context of mucinous histology. The NCCN guidelines similarly advocate for comprehensive staging surgery in cases of clear cell carcinoma, including systematic lymphadenectomy. In confirmed or suspected mucinous ovarian carcinoma, appendectomy is recommended only if macroscopically abnormal. The BGCS provides a more conservative perspective regarding lymphadenectomy in specific histological subtypes. In particular, in expansile-type mucinous tumors and low-grade endometrioid carcinomas, systematic lymph node dissection may not be warranted due to the low risk of node metastasis. Appendectomy may be considered when mucinous histology is suspected, although the diagnostic yield is relatively low in the absence of macroscopic abnormalities. However, if appendiceal pseudomyxoma peritonei is identified during staging laparotomy, definitive surgical management is indicated. The Japanese guidelines do not address this topic and do not provide further specifications based on epithelial histological subtype.

### 3.5. First Line Chemotherapy

#### 3.5.1. Low Grade Serous Ovarian Cancer

LGSOC represents a rare and distinct subtype of epithelial ovarian cancer, characterized by relative resistance to conventional chemotherapy and a prolonged, indolent clinical course. The management of advanced and recurrent LGSOC has evolved significantly over the past decade, integrating both cytotoxic and hormonal therapies, as well as targeted agents. Notably, in the recurrent setting, the MEK inhibitor trametinib has demonstrated higher response rates compared to second-line chemotherapy or endocrine therapy—6% versus 26% in the GOG 281/LOGS trial [68]—and is now considered a viable option following prior platinum-based treatment. This represents a paradigm shift in the systemic management of recurrent LGSOC, where cytotoxic agents often demonstrate limited efficacy. Currently, Trametinib is suggested by NCCN, BGCS and ESMO-ESGO guidelines.

According to the most recent guidance from the BGCS, a platinum–taxane regimen may be offered to patients with advanced disease.

The NCCN provides more granular recommendations based on stage and histology. For patients with early-stage LGSOC, the preferred approach includes observation for stages IA and observation versus carboplatin and paclitaxel administered every three weeks, with the optional addition of maintenance hormonal therapy such as letrozole, for stages IB-IC. Hormonal monotherapy with aromatase inhibitors (anastrozole, letrozole, exemestane) is also acceptable. For advanced-stage disease (stage II–IV), NCCN maintains platinum–taxane chemotherapy as a preferred option, optionally combined with bevacizumab and followed by maintenance therapy with bevacizumab or hormonal agents. In the setting of recurrence, NCCN guidelines consider secondary cytoreduction in case of oligo -, well-isolated recurrence and/or systemic therapy such as chemotherapy, if not previously used, and hormonal therapy; fulvestrant is suggested as an endocrine-based option. Depending on the molecular profiling of recurrent disease, dabrafenib (a BRAF inhibitor) can be added to trametinib in BRAF V600E-positive patients and avutometinib + defactinib or binitinib alone (MAPK-pathway directed drugs) can be an option in KRAS-mutated patients.

According to ESMO–ESGO consensus, in stage II disease, adjuvant chemotherapy may be considered, potentially followed by maintenance endocrine therapy. For stages III and IV, chemotherapy with paclitaxel and carboplatin—with or without bevacizumab—remains an acceptable option. Endocrine maintenance therapy following first-line chemotherapy is emphasized as a reasonable strategy in the absence of progression. In cases of relapse, trametinib is identified as a promising therapeutic alternative, particularly after failure of prior platinum-based or hormonal treatment. Consistent with these perspectives, the ESMO guidelines explicitly state that adjuvant chemotherapy is not recommended for patients with stage IA LGSOC and remains optional for stages IB and IC. As highlighted in Table 3, there is a notable divergence among guidelines concerning adjuvant therapy recommendations for low-grade serous carcinoma, particularly in the context of stage I and its substages.

Collectively, these guidelines reflect a growing consensus around the limited efficacy of cytotoxic chemotherapy in LGSOC and a shift toward incorporating endocrine therapies and targeted agents earlier in the treatment pathway. While chemotherapy remains a foundational option in the first-line setting—especially in advanced stages—the use of maintenance hormonal therapy is increasingly supported across guidelines, mirroring treatment strategies seen in hormone receptor–positive breast cancer. The integration of MEK inhibitors, particularly trametinib, marks a significant advancement in the management of recurrent disease and highlights the importance of molecularly targeted approaches in this patient population. Nevertheless, variation persists in guideline recommendations, especially regarding the role and timing of chemotherapy in early-stage disease, underscoring the need for continued prospective trials to guide individualized treatment strategies for LGSOC.

#### 3.5.2. Borderline Tumors

International guidelines mostly agree on advocating a conservative approach, although specific recommendations differ based on histopathological features like the presence of invasive implants or residual disease.

The BGCS clearly opposes the use of cytotoxic chemotherapy in managing BOTs, stating that there is currently no evidence-based reason to use it. This position reflects the low recurrence rates and excellent long-term outcomes usually seen in patients with fully removed borderline tumors, especially in early-stage cases. Similarly, the Australian guidelines recommend against adjuvant chemotherapy unless invasive peritoneal implants are confirmed through histological examination. Likewise, NCCN advises customizing postoperative treatment based on whether invasive peritoneal implants are present or not. For patients with invasive implants, postoperative chemotherapy might be considered, using the same regimens as those used for low-grade serous ovarian carcinoma. The Japanese guidelines are consistent with NCCN’s recommendations. The ESMO–ESGO consensus guidelines do not offer specific advice regarding systemic therapy for BOTs (Table 3).

In summary, while most international guidelines do not support routine use of chemotherapy in BOTs, a more selective approach is recommended when there are adverse pathological features such as invasive peritoneal implants or residual tumor. The lack of a unified international consensus highlights the need for ongoing research into prognostic markers and treatment strategies that can better inform decisions for this particular subset of ovarian tumors.

#### 3.5.3. Early Stages of Epithelial Ovarian Cancer

Most guidelines recommend adjuvant platinum-based chemotherapy for early-stage EOC, especially for tumors with high-grade histology or unfavorable subtypes (Table 4). The standard regimen includes paclitaxel and carboplatin given every three weeks, although alternatives like carboplatin with liposomal doxorubicin or docetaxel are also acceptable. While six cycles are typically the standard for most high-risk patients, shorter regimens (three cycles) may be suitable for certain low-risk non-serous subtypes.

There is a consensus that both histologic subtype and tumor grade are key factors in determining the benefit of adjuvant chemotherapy. High-grade serous and grade 3 endometrioid tumors are consistently regarded as indications for chemotherapy across all guidelines. Conversely, clear cell and mucinous tumors are known to be less responsive to chemotherapy, resulting in more tailored treatment approaches. For clear cell carcinoma, recommendations differ. While BGCS and ESMO-ESGO permit omitting chemotherapy in completely staged IA/IB disease, NCCN recommends treatment regardless of stage. For mucinous carcinoma, the infiltrative sub-types, particularly stage IB–IC3, are considered for adjuvant therapy, especially according to ESMO and NCCN; these guidelines clearly outline stage-specific considerations and distinguish between expansile and infiltrative subtypes as important factors to consider when determining adjuvant therapy. For low-grade endometrioid carcinomas (stage IA/IB), all guidelines agree that chemotherapy can be safely omitted when there is complete surgical staging.

#### 3.5.4. Advanced Stages Epithelial Ovarian Cancer

Across international guidelines, a platinum-based doublet remains the standard of care for patients with advanced-stage disease, most commonly a combination of carboplatin (AUC 5–6) and paclitaxel (175 mg/m^2^) administered intravenously every 3 weeks for six cycles. For patients who are intolerant to paclitaxel—due to allergy, neuropathy, or other significant toxicities—all guidelines suggest alternatives such as docetaxel or pegylated liposomal doxorubicin in combination with carboplatin. (Table 5). Updated meta-analysis of randomized clinical trials [69] did not find PFS superiority of the dose-dense schedule when compared to the three-weekly schedule. Weekly dosing schedules (e.g., weekly paclitaxel 60–70 mg/m^2^ with carboplatin AUC 2) may be considered for frail or elderly patients unable to tolerate standard three-weekly regimens, as highlighted particularly by the BGCS.

A key area of interest among the guidelines concerns the use of bevacizumab in the first-line setting [70,71]. Several guidelines—including those from BGCS, ESMO-ESGO, the Japanese group, and the Australian recommendations—support adding bevacizumab to the standard carboplatin–paclitaxel regimen in selected high-risk patients, especially those with stage III disease with macroscopic residual tumor or stage IV disease. Bevacizumab may be continued as maintenance therapy. A Cochrane review published in 2023 [72], however, found that bevacizumab, given with chemotherapy and continued as maintenance, likely results in little difference in OS compared to chemotherapy alone, thus highlighting an uncertain role for this chemotherapeutic agent in newly diagnosed advanced ovarian cancer. The ESMO-ESGO and NCCN guidelines emphasize that the use of bevacizumab should not be guided by the presence of molecular biomarkers such as BRCA mutations or homologous recombination deficiency, as current evidence does not support restricting its use based on genetic profiles, since BRCA status does not significantly modify the effect of bevacizumab on PFS [73]. The NCCN guidelines also urge caution when using bevacizumab in the neoadjuvant setting, citing potential interference with postoperative wound healing. They recommend withholding the drug for 4–6 weeks before IDS and delaying its reintroduction after surgery to reduce the risk of complications such as fistula or dehiscence. BGCS also echoes these safety considerations.

Several ongoing clinical trials are investigating the role of immunotherapy in advanced ovarian cancer (i.e., pembrolizumab and nivolumab are anti-PD-1 monoclonal antibodies; avelumab, atezolizumab, and durvalumab inhibit PD-L1) [74]; at present date, none of the major international guidelines have incorporated immunotherapy into their standard treatment recommendations for advanced ovarian cancer. Further data are awaited to better define the potential role of these agents in the first-line or maintenance setting.

### 3.6. Maintenance Systemic Treatment

While the role of maintenance therapy is well established, especially in patients who achieve a complete or partial response after platinum-based chemotherapy, international guidelines show both agreement and differences regarding recommended strategies. Molecular characteristics of the tumor are a critical factor in guiding the choice of maintenance therapy following first-line treatment, as existing literature demonstrates that BRCA and HRD mutational status influence treatment response, OS, and PFS [41]. All guidelines explicitly require homologous recombination assessment prior to maintenance therapy, especially when considering PARP inhibitors. Recommendations from major guidelines are summarized in Table S7.

The BGCS recommends maintenance treatment with bevacizumab in combination with chemotherapy and as a single agent thereafter. In patients with stage III–IV disease, the use of PARP inhibitors, particularly olaparib, should be considered following a response to first-line chemotherapy. In those with HRD, maintenance with olaparib in combination with bevacizumab is advised. Similarly, the NCCN guidelines support single-agent bevacizumab maintenance therapy for patients with stage II–IV disease who respond or achieve stable disease after receiving a bevacizumab-containing chemotherapy regimen. However, the NCCN no longer recommends bevacizumab monotherapy as a maintenance strategy in patients with BRCA1-2 mutations, while it remains a viable option for those with wild-type or unknown BRCA status. Olaparib monotherapy is specifically recommended for patients with BRCA1-2 mutations.

In contrast, the combination of olaparib and bevacizumab may be offered to patients regardless of HRD status, provided they received bevacizumab during first-line treatment. Niraparib is indicated for patients with BRCA wild-type or unknown status if they achieved a response following chemotherapy without bevacizumab, or in BRCA-mutated patients who received bevacizumab. Notably, veliparib has been investigated in this setting [75] but is not US Food and Drug Administration (FDA)-approved. This distinction is noted by the NCCN, which also cautions about discrepancies between FDA approvals and NCCN recommendations—differences that may contribute to clinical uncertainty in practice. To date, olaparib, rucaparib, and niraparib have all obtained FDA and European Medicines Agency (EMA) approval in ovarian cancer as maintenance therapy both in primary and recurrent settings.

Japanese guidelines adopt a more conservative approach. Maintenance therapy is generally not recommended for patients who achieve complete remission after first-line chemotherapy, unless bevacizumab was part of the initial regimen. In those cases, continuation of bevacizumab maintenance may be considered. Additionally, olaparib is recommended for patients with BRCA1-2 mutations who reach complete remission after chemotherapy, reflecting a more selective application of maintenance therapy based primarily on BRCA status and prior treatment exposure.

The ESMO guidelines offer a more stratified and biomarker-driven framework. It suggests that maintenance treatment with PARP inhibitors, with or without bevacizumab, should be offered to patients with BRCA-mutated or HRD-positive tumors who achieve a response or show no evidence of disease following platinum-based chemotherapy. Specifically, olaparib is recommended for two years in BRCA-mutated patients, either alone or in combination with bevacizumab, while niraparib is indicated for three years. In patients with BRCA wild-type/HRD-positive tumors, both niraparib (three years) and olaparib-bevacizumab (two years) are valid options. For HRD-negative patients, either bevacizumab alone or niraparib (for three years) can be considered. The ESMO-ESGO consensus further refines these recommendations by suggesting that patients who are BRCA wild-type and HRD-negative may receive bevacizumab maintenance after chemotherapy or may be offered PARP inhibitors such as niraparib or rucaparib. However, the option of no maintenance treatment is also recognized. For those with unknown HRD status, both bevacizumab-based and PARPi-based maintenance strategies remain acceptable.

In conclusion, despite a shared recognition of the importance of maintenance strategies in extending disease control in advanced ovarian cancer, differences remain in patient selection criteria, biomarker thresholds, and choice of agents. These variations reflect differences in regulatory approvals, national drug availability, and interpretation of key clinical trials. Harmonizing these approaches while maintaining flexibility for individual patient situations remains a key challenge.

### 3.7. HIPEC

The use of hyperthermic intraperitoneal chemotherapy (HIPEC) in epithelial ovarian cancer has sparked significant interest over the past two decades. However, its integration into standard clinical practice remains inconsistent across international guidelines, reflecting ongoing uncertainty and variation in clinical evidence.

According to BGCS, HIPEC may be considered during IDS for patients with newly diagnosed ovarian cancer, in line with the NICE interventional procedure guidance. The NCCN guidelines suggest that intraoperative HIPEC may be beneficial when performed at the time of IDS in patients who have shown either a response or stable disease after NACT, including stage IV patients who respond to NACT or whose stage IV disease sites have resolved or are considered resectable. In this context, NCCN cites a commonly used regimen consisting of intraoperative cisplatin at a dose of 100 mg/m^2^. The guidelines clearly state that HIPEC is not recommended in primary debulking surgery, where benefits have not been proven and potential risks may outweigh any therapeutic gains. Conversely, both the Japanese and European guidelines take a much more cautious stance, recommending HIPEC only in an experimental setting. The ESMO–ESGO guidelines explicitly advise against using HIPEC during cytoreductive surgery for relapsed ovarian cancer.

Taken together, these guideline positions highlight a lack of international consensus on the role of HIPEC in ovarian cancer treatment. While the NCCN and BGCS permit its use under strict conditions—restricted to IDS following NACT and performed at specialized centers—the ESMO, ESGO, and Japanese guidelines limit its use to investigational settings only. All groups, however, agree on the need for more high-quality evidence, ideally through large-scale randomized trials, to clarify the real clinical benefit, suitable patient selection criteria, and potential risks of HIPEC. Until such data are available, the use of HIPEC should be limited to expert centers with proper infrastructure, multidisciplinary support, and rigorous clinical oversight.

### 3.8. Recurrence

A complex interplay of clinical and biological factors influences the selection of chemotherapy for relapsed epithelial ovarian cancer. These include the patient’s performance status and preferences, residual toxicities from prior treatments, history of hypersensitivity reactions, and the timing and nature of previous responses to platinum-based chemotherapy. Historically, a platinum-free interval (PFI) greater than 6 months has been used to categorize patients as “platinum-sensitive.” According to the latest Ovarian Cancer Consensus Conference [76], PFI should be replaced by the broader treatment-free interval (TFI), which can include TFI from the last platinum dose (TFIp), TFI from the last non-platinum therapy (TFInp), as well as TFI from the last biological agent (TFIb), especially important in the era of targeted and maintenance therapies. Treatment decisions should not rely solely on rigid time-based classifications, as evidenced by the fact that patients with a TFIp less than 6 months may still respond to platinum-based combinations, particularly when other factors such as tumor histology or BRCA1-2 mutation status are considered. In fact, BRCA mutation status is a key example of how molecular biomarkers can more accurately stratify patients than TFIp alone.

Given the increasing complexity of the therapeutic landscape, participation in clinical trials is essential, especially for patients with recurrent disease who may not clearly fit into traditional categories of platinum-sensitive or platinum-resistant. Clinical trials offer access to new agents and combinations and are vital for discovering effective treatments in molecularly defined subgroups. Particularly when standard options are limited or prior lines of therapy have failed, enrollment in clinical studies is recommended by all international guidelines.

Similarly, all international guidelines agree on the role of secondary cytoreductive surgery as a viable option that may provide a survival benefit if complete cytoreduction is possible [77,78]. In contrast, for patients with platinum-resistant disease, secondary surgery is generally not recommended, except in palliative settings for symptom relief or when a solitary resectable lesion is present.

In patients with platinum-sensitive recurrence, all major international guidelines recommend re-treatment with platinum-based combination regimens. The BGCS advocates for combination regimens as the preferred approach. Similarly, the NCCN recommends six cycles of platinum-based doublets such as carboplatin/gemcitabine, carboplatin/paclitaxel, or carboplatin/liposomal doxorubicin, with or without bevacizumab. Japanese guidelines also support platinum-based combinations in this setting. ESMO-ESGO guidelines further endorse platinum rechallenge in platinum-sensitive patients, highlighting the combination of carboplatin and pegylated liposomal doxorubicin as the preferred option. ESMO emphasizes that patients with platinum intolerance who relapse after more than six months may benefit from trabectedin in combination with liposomal doxorubicin.

For patients with platinum-resistant disease, all guidelines recommend shifting to non-platinum-based strategies. Except for the NCCN, which outlines a wide range of non-platinum combination options, all guidelines prefer single-agent chemotherapy in this context, citing comparable efficacy and lower toxicity compared to combination regimens. The NCCN includes weekly paclitaxel, liposomal doxorubicin, gemcitabine, oral etoposide, topotecan, and capecitabine, with or without bevacizumab, depending on patient tolerance and prior exposure.

Bevacizumab plays a key role in managing recurrent ovarian cancer in both platinum-sensitive and platinum-resistant settings, although its use varies slightly among guidelines based on patient-specific factors and previous treatments. The NCCN recommends using bevacizumab alone or combined with both platinum-based and non-platinum regimens and supports continuing it as maintenance therapy until disease progression or unacceptable toxicity occurs. Preferred combinations include weekly paclitaxel, liposomal doxorubicin, or topotecan with bevacizumab. Japanese guidelines endorse continuing bevacizumab in patients who previously received it with chemotherapy and have platinum-resistant disease. ESMO-ESGO adopts a more nuanced approach. For patients previously treated with bevacizumab in the first-line setting, rechallenge with bevacizumab may be considered upon recurrence. For those previously treated with PARP inhibitors but not bevacizumab, a platinum-based combination with bevacizumab followed by maintenance is recommended. In BRCA wild-type or BRCA-unknown patients with no prior exposure to PARPi or bevacizumab, maintenance therapy with either a PARPi or bevacizumab is advised. ESMO supports its use in combination with both platinum and non-platinum regimens, followed by maintenance, and recommends continuing until disease progression.

The integration of PARP inhibitors into the management of relapsed ovarian cancer has become standard for patients with BRCA-mutated or HRD-positive tumors, especially those who respond to platinum-based rechallenge. Both BGCS and ESMO-ESGO recommend PARPi maintenance for patients who are PARPi-naïve. The NCCN supports their use if there has been no prior progression on PARP therapy and provides detailed guidelines: olaparib is approved for germline BRCA-mutated patients after three or more prior lines of therapy; rucaparib is preferred in platinum-resistant BRCA-mutated patients due to limited options; niraparib is recommended for platinum-sensitive recurrence after at least two prior platinum regimens. Japanese guidelines also endorse olaparib for BRCA-mutated patients following response to platinum rechallenge. ESMO-ESGO elaborates on rechallenge strategies, suggesting PARPi re-treatment in patients with extended prior PARPi exposure (≥18 months in first-line or ≥12 months in later lines for BRCA-mutated cases). Furthermore, in patients with no previous PARPi exposure, maintenance therapy with any PARPi is advised, regardless of BRCA or HRD status. ESMO 2023 confirms that olaparib, niraparib, and rucaparib are approved regardless of mutational status in patients responding to platinum-based therapy, and recommends continuing until disease progression or unacceptable toxicity.

Overall, all major international guidelines agree on the key role of platinum-based therapy in platinum-sensitive recurrence and the use of non-platinum agents in resistant cases, with increasing focus on maintenance strategies involving PARP inhibitors and bevacizumab based on prior treatment history and molecular profiles. (Table 6).

### 3.9. Radiotherapy

According to NCCN guidelines, radiotherapy (RT) is not recommended as part of the first-line treatment for epithelial ovarian cancer. This is because of the proven effectiveness of cytoreductive surgery and systemic chemotherapy, along with the lack of supporting evidence for RT in the initial setting. In the guidelines, RT is only suggested in specific scenarios: oligometastatic relapse with slow progression and localized lesions in patients who are unfit for surgery, or for palliative purposes.

Similarly, the European guidelines ESGO and ESMO do not include RT in the standard therapeutic pathway for early-stage disease. However, they recognize a potential role in selected clinical settings or within experimental protocols, especially with modern techniques such as Stereotactic Body Radiation Therapy (SBRT). Although not routinely recommended, RT may be considered for isolated recurrences, symptomatic patients, and patients enrolled in clinical trials. Additionally, AOIM confirms the marginal role of RT in first-line therapy. This guideline offers a detailed overview of new opportunities provided by advanced techniques. Notably, it emphasizes the potential of SBRT, which is ideal for small, well-defined lesions and was initially used for brain metastases but has now been extended to nodal and visceral lesions.

All reviewed guidelines agree on one point: RT may be considered in specific scenarios, such as unresectable oligometastatic disease, localized recurrences, and palliative treatment for local symptom relief (pain, bleeding, compressions) (Table S8). SBRT enables the precise delivery of high-dose radiation beams over a short period and in a few sessions, aiming for a radical cure. It may offer a targeted approach in recurrent oligometastatic disease due to its high local control, minimal toxicity, and low complication rates, potentially delaying systemic cytotoxic treatments without significantly affecting survival. The multicenter retrospective study MITO-RT1 is currently the largest dataset available in the literature on SBRT in patients with oligometastatic ovarian cancer [79]. It includes 261 patients treated for 449 lesions and, thanks to highly conformal, image-guided techniques, showed a 2-year local PFS of 81.9% and an OS of 73.6%. Regarding toxicity, it was mainly late grade 1 and 2, with a 2-year late toxicity-free survival of 95.1% and low-grade acute toxicity in 20.6% of cases.

### 3.10. Fertility Sparing

In recent decades, fertility preservation for young women with ovarian cancer has become increasingly important, especially with advances in surgical techniques and the rising average age at which women conceive. Major international guidelines have gradually incorporated recommendations for fertility-sparing surgery (FSS), although there are notable differences regarding indications, selection criteria, and surgical methods.

The new joint guidelines by ESGO, European Society of Human Reproduction and Embryology (ESHRE) and European Society for Gynaecological Endoscopy (ESGE) give particular attention to fertility preservation in women with ovarian cancer, acknowledging that an increasing number of young patients wish to maintain their potential for pregnancy even after a cancer diagnosis. In this guideline, fertility preservation is recommended for patients with non-invasive borderline ovarian tumors of any stage and for all types of germ cell tumors, which generally have high chemosensitivity and a favorable prognosis. It is also considered appropriate in granulosa cell tumors stage IA and IC1, in well or moderately differentiated Sertoli-Leydig cell tumors stage IA, and in some low-grade epithelial carcinomas, such as serous and endometrioid types in stage IA–IC1, as well as in expansile mucinous carcinomas and clear-cell carcinomas at an early stage. In selected cases, fertility preservation may also be evaluated in stage IC2 or IC3 tumors, provided that the tumor biology is favorable and the patient is followed in a highly specialized center. Guidelines advise against lymphadenectomy in mucinous ovarian cancer. Fertility preservation is not recommended for invasive epithelial ovarian cancers stage IB or higher, high-grade serous or endometrioid carcinomas, clear-cell carcinomas stage IC3, and small-cell carcinomas of the hypercalcemic type, all associated with a high risk of recurrence.

The AIOM guidelines recognize conservative surgery as the standard for BOTs, which predominantly affect young women. Fertility preservation surgery is accepted for high-grade serous carcinoma stage IA or IC1, low-grade papillary serous carcinoma IA-IC, endometrioid carcinoma IA–IC, expansive mucinous tumors IA–IC, and clear cell carcinoma IA. Conservative intervention involves preserving the uterus and at least one ovary, combined with comprehensive surgical staging, including omentectomy, peritoneal cytology, multiple biopsies, and, in mucinous tumors, appendectomy. In selected cases (e.g., bilateral involvement), preservation of the uterus alone may be considered for future assisted reproductive technologies. The BGCS emphasizes early identification of FSS-eligible patients through a multidisciplinary team, recommending individualized assessment and comprehensive counseling regarding oncological and reproductive implications. Conservative surgery is allowed in unilateral stage IA or IC tumors with favorable histotypes (G1–G2 serous, mucinous, endometrioid, or mixed) and BOTs. The ESGO-ESMO recommendations align in recognizing the safety of FSS in BOTs, in low-grade epithelial tumors at stage IA–IC1, and in selected IC2 cases, provided there is favorable histology and unilateral involvement. Minimally invasive surgery is accepted if capsular rupture is avoided. According to ESMO guidelines for early-stage epithelial tumors, including FIGO IA–IC1–IC2, fertility-sparing surgery may be considered in low-grade subtypes (serous, endometrioid, and expansile mucinous) following complete staging and only if there is no extraovarian involvement. The risk of upstaging after surgical staging is particularly emphasized, with significant prognostic and therapeutic implications. Laparotomy remains the standard approach according to ESMO. The NCCN guidelines offer similar recommendations, stating that FSS may be provided to patients with stage IA–IC1 epithelial tumors and BOTs. Even in bilateral cases (stage IB), uterine preservation may be considered for future assisted reproduction. FSS is generally not recommended for clear cell tumors. The Sub-Saharan NCCN-aligned guidelines mirror those of the American guidelines, with particular emphasis on staging and follow-up, and the possibility of performing FSS even in low-risk tumors using a minimally invasive approach in selected cases. Japanese guidelines support FSS for non-clear cell epithelial carcinomas at stage IA–IC1 and low grade, while the same approach is accepted for clear cell tumors only at stage IA. Australian guidelines do not specify recommendations for FSS in ovarian cancer.

Overall, all guidelines agree on the appropriateness of conservative surgery in low-risk early-stage ovarian tumors, with broad consensus for BOT and unilateral low-grade epithelial subtypes (G1–G2). (Table S9) However, differences remain regarding oncological safety thresholds, the use of laparoscopy, inclusion of clear cell tumors, and staging strategies. A shared emphasis is placed on personalized, multidisciplinary counseling that considers prognosis, reproductive goals, and available options, ensuring a truly informed and individualized approach for each patient.

### 3.11. Follow-Up Strategies

The goals of follow-up include identifying and managing treatment-related toxicities, supporting patients’ psychosocial and reproductive health, and promoting preventive strategies such as screening for secondary cancers and genetic counseling. At the same time, the survival benefit of intensive follow-up strategies remains debated. Randomized studies like the MRC OV05/EORTC 55955 trial [80] have shown that early detection of recurrence, especially through the use of serum markers, does not improve overall survival, which questions the value of overly frequent or aggressive monitoring. International guidelines show both similarities and differences in their approach to post-treatment surveillance. (Table S10)

#### 3.11.1. Role of CA-125

Among biomarkers, CA-125 has historically been the most widely used for ovarian cancer follow-up. Its levels often correlate with disease recurrence, typically rising two to five months before clinical or radiological relapse. The Italian AOIM guidelines highlight its high sensitivity and specificity, suggesting it can help identify patients who might benefit from further diagnostic testing. However, isolated biochemical recurrence alone is not enough to justify restarting chemotherapy, and patients without radiological or symptom evidence are not currently candidates for treatment. Recently, analysis of the SOLO 2 trial [81] data showed that CA-125 levels during maintenance therapy with olaparib in BRCA-mutated patients have low agreement with imaging studies in detecting disease progression.

The BGCS guidelines are more cautious and explicitly reference the randomized MRC/EORTC trial, which demonstrated no survival difference between patients treated at the time of biochemical progression and those treated once symptoms appeared. Moreover, patients in whom treatment was started early based on CA-125 rise alone experienced earlier deterioration in global health scores, underscoring the potential harm of such an approach. Today, therefore, it is now accepted that a rising CA125 alone, without clinical or radiographic evidence of recurrence, should not routinely be used as an indication to commence systemic chemotherapy. The NCCN guidelines and the Sub-Saharan African guidelines consider CA-125 monitoring to be optional and advise that it should only be used when elevated at diagnosis. Importantly, they recommend that patients be counseled about the benefits and limitations of CA-125 surveillance, as some may find reassurance in close monitoring while others may prefer to avoid the anxiety associated with serial testing. The benefit of surveillance with CA-125 in the current era when more sensitive radiological detection methods such as PET/CT and complete secondary cytoreduction and targeted therapy have been shown to improve outcomes has yet to be defined. Japanese and Australian guidelines adopt a pragmatic approach, acknowledging that CA-125 is valuable when elevated at baseline but cautioning against using it as the sole determinant for therapeutic intervention.

Overall, the consensus across guidelines is that CA-125 has a role in surveillance but should not by itself dictate therapeutic decisions (Table S11). Clinical and radiological confirmation remains essential before resuming treatment.

#### 3.11.2. Role of Imaging

The role of imaging during follow-up is equally debated. The AOIM, referencing American College of Radiology (ACR) criteria, considers contrast-enhanced CT scans to be the reference imaging modality, with high sensitivity and specificity for detecting recurrence. PET/CT is also considered appropriate although it is less reliable in certain histological subtypes such as mucinous and clear cell carcinomas. All patients undergoing conservative treatment must undergo periodic transvaginal ultrasound. In contrast, the NCCN and ESGO-ESMO do not recommend routine imaging in asymptomatic patients, reserving scans for cases in which symptoms, abnormal clinical findings, or suspicious biomarker elevations raise concern for relapse. This approach is supported by data showing that routine imaging does not confer a survival benefit and may expose patients to unnecessary radiation, cost, and anxiety. The Japanese guidelines are particularly restrictive, discouraging the routine use of CT, MRI, or PET during surveillance and limiting their application to situations of clinical or laboratory suspicion. They do, however, recognize the potential utility of transvaginal or abdominal ultrasound in selected patients, especially those who have undergone fertility-sparing surgery.

Thus, while imaging remains a cornerstone in the detection of symptomatic recurrence, most international guidelines agree that its routine use in asymptomatic women is not justified.

#### 3.11.3. Frequency of Follow-Up Visits

One of the most practical aspects of follow-up is determining how frequently patients should be evaluated. In this context, international guidelines demonstrate a degree of convergence. The majority of them recommend visits every two to four months during the first two years, when the risk of recurrence is highest. The interval is then extended to every three to six months for years three to five, followed by annual visits thereafter. The BGCS guidelines adopt a similar schedule but recommend every three months for the first two years and every six months up to five years.

This harmonization of schedules across guidelines reflects the shared understanding that the early years after treatment carry the greatest risk of recurrence, warranting closer observation.

#### 3.11.4. Survivorship and Long-Term Care

Beyond recurrence detection, all guidelines emphasize the importance of survivorship care as an integral component of follow-up. The ESGO-ESMO guidelines advocate for systematic assessment of quality of life and symptoms using validated tools, with early integration of psycho-oncology, social care, and supportive services. Similarly, NCCN and Sub-Saharan African guidelines highlight survivorship programs and wellness care, stressing the importance of genetic counseling for patients who have not previously undergone testing. BGCS and Australian guidelines also emphasize holistic follow-up, highlighting the importance of addressing psychosocial, emotional, and sexual health issues. They advocate for clear communication pathways, ensuring that patients have rapid access to their healthcare team in the event of new or unexpected symptoms.

#### 3.11.5. Liquid Biopsy

Circulating tumor DNA (ctDNA) and other liquid biopsy methods are being studied and may, in the future, offer more accurate tools for monitoring minimal residual disease and guiding treatment decisions [19]. Major international guidelines do not yet support the routine use of ctDNA or liquid biopsy to replace CA-125 or imaging in standard ovarian cancer follow-up; its use is primarily recommended in research settings or clinical trials. Potential roles include early detection of recurrence: studies indicate that ctDNA can identify molecular recurrence before traditional methods in some patients, but there is no solid evidence that this early detection improves survival unless the treatment plan is effectively adjusted. However, recent guidelines highlight the limitations of this practice due to variability among studies and the lack of randomized trials showing a clear clinical benefit from ctDNA monitoring during follow-up. Therefore, the current clinical advice is cautious. ESGO guidelines state that routine monitoring of ctDNA is not recommended. NCCN guidelines note that there is no general indication for using ctDNA in routine follow-up outside of research studies. Additionally, ESMO guidelines have published recommendations on the overall role of ctDNA in oncology practice, stressing its usefulness and potential in many areas, but routine use requires clinical evidence demonstrating its utility for specific applications.

The follow-up of epithelial ovarian carcinoma remains an area of uncertainty and variability. Although international guidelines differ in their specific recommendations, several points of agreement have emerged. Clinical evaluation and symptom assessment continue to be the foundation of surveillance. CA-125, while sensitive, should not be the sole factor determining treatment decisions, and imaging should be used only for symptomatic patients or those with suspicious findings. The frequency of follow-up visits is highest in the first two years and gradually decreases afterward. Importantly, survivorship care—including the management of late toxicities, genetic counseling, psychological support, and lifestyle promotion—is increasingly seen as a crucial part of follow-up.

In conclusion, follow-up after primary treatment of ovarian cancer should be personalized, multidisciplinary, and patient-centered, combining oncological caution with long-term quality-of-life maintenance. As treatment options continue to advance and change the course of the disease, follow-up strategies will also need to adapt, balancing the advantages of early detection of recurrence with the risks associated with overmedicalization and unnecessary procedures.

## 4. Discussion

This review provides a narrative synthesis of the current recommendations from the major clinical practice guidelines on the management of ovarian cancer. As a consequence, it is subject to an inherent temporal heterogeneity, as each guideline is updated according to its own schedule and with varying degrees of responsiveness to new evidence. A time-related bias may arise, reflecting discrepancies between when new data become available and when they are formally incorporated into guideline revisions. The rapid evolution of ovarian cancer research—encompassing advances in molecular profiling, surgical techniques, and systemic therapeutics—implies that emerging evidence and promising novel agents may not yet be fully incorporated into the most recently published guideline editions. Accordingly, even a thorough synthesis of existing recommendations can represent only the status of guideline-directed management at the specific time of review.

One of the main takeaways from this review is that clinical management of patients needs to shift from a one-size-fits-all approach to a more personalized strategy, considering tumor histotype, stage, molecular profile, and patient-specific factors like age, reproductive goals, and performance status. So far, these differences have led to only minor variations in treatment protocols. However, achieving the best therapeutic results increasingly depends on accurate patient stratification. New clinical trials are gradually addressing tumor heterogeneity by using subgroup classifications or specific biomarkers as inclusion criteria, recognizing their influence on treatment response and trial success. In particular, molecular and immunohistochemical tumor profiling is an emerging area of research, especially in the context of targeted therapies [82].

A standardized global approach to ovarian cancer treatment still faces major challenges due to sociocultural, economic, and systemic differences embedded in clinical guidelines, drug approval policies, and healthcare infrastructures across countries. However, fostering international collaboration, promoting equal access to innovative therapies, and harmonizing treatment strategies through shared evidence may be crucial steps toward improving outcomes for all patients with ovarian cancer. Centralization of ovarian cancer care should be regarded as a key quality indicator in modern oncologic practice. Management within high-volume, specialized centers—with access to multidisciplinary expertise and advanced surgical and molecular diagnostics—correlates with improved survival outcomes and better adherence to guideline-recommended therapies. Encouraging referral to certified centers of excellence not only ensures consistency in clinical decision-making but also facilitates patient inclusion in clinical trials and access to novel therapeutic options. In this context, future guideline updates should explicitly emphasize the role of centralization and certification processes as essential components of an equitable and effective ovarian cancer care model.

## 5. Conclusions

This narrative review provides a comprehensive overview of the most recent published guidelines on ovarian cancer management, highlighting discrepancies and variations that reflect the disease’s inherent complexity and heterogeneity. Each section carefully examines both similarities and differences in managing ovarian cancer across all stages, from prevention to recurrence. Comparing these guidelines reveals several key considerations for future updates.

## 6. Future Directions

The concept of personalized medicine has profoundly reshaped the clinical management of ovarian cancer. Understanding and predicting individual responses to chemotherapy has become a critical objective in both translational and clinical research to achieve better prognosis in patients with EOC.

Preclinical studies provide an essential platform for elucidating the molecular mechanisms underlying chemotherapy sensitivity and resistance. Through the use of in vitro and in vivo models—such as patient-derived cell lines, organoids, and patient-derived xenografts—researchers can investigate drug–tumor interactions under controlled conditions that reflect patient-specific tumor biology. Patient-derived organoids (PDO) are particularly promising as they maintain genetic stability and tumor heterogeneity while being less time-consuming and expensive. These models enable the exploration of biological and molecular features of tumors to elucidate the mechanisms underlying tumor growth, progression, and therapeutic response. A deeper understanding of the tumor microenvironment, particularly its immune components such as B cells and mast cells, may help uncover pathways of immune suppression and aid in the identification of predictive and prognostic biomarkers. Several studies [83,84] used PDO cultures to assess the concordance between in vitro platinum sensitivity and clinical outcomes, as well as to characterize tumor-infiltrating immune populations potentially associated with prognosis and several clinical trials are (NCT02732860, NCT04279509, NCT04768270, NCT05175326, NCT05290961). Nevertheless, several challenges still remain. In particular, the lack of standardization across model systems and organoid culture development, patient group heterogeneity and biopsy variability limit the direct applicability of laboratory findings into clinical practice. Future research is needed to overcome present limitations and to test the consistency of the organoid drug screening model. Functional assays in PDO models, integrated with molecular profiling and clinicopathological parameters, represent however a promising tool to study tumor pathobiology and to obtain real-time, preclinical, prediction of chemotherapeutic response that could help clinicians in individualized therapeutic choices.

Future directions are closely linked to the integration of artificial intelligence (AI) into ovarian cancer management and, more broadly, gynecological oncology. AI applications span multiple domains, including the development of detection tools leveraging omics sciences, the improvement of diagnostic accuracy in differentiating ovarian masses, and the enhancement of surgical performance. In surgery, AI contributes both technically—through the recognition of anatomical structures, suspicious lesions, and laparoscopic instruments, leading to improved surgical outcomes and reduced complication rates—and predictively, by estimating the likelihood of complete cytoreduction, therapeutic response, and hospital stay. This rapidly evolving field is supported by an expanding body of literature encompassing screening, diagnostics, surgery, histopathology, oncology, and prognostic evaluation, underscoring the growing impact of AI across gynecological oncology. Current evidence supports its role as a complementary clinical and research tool for diagnosis, patient stratification, and prediction of histopathological correlations in gynecological malignancies [85,86,87,88]. However, challenges related to machine learning errors, safety, and transparency in regulatory approval processes remain to be addressed.

Strengthening the connection between preclinical and clinical research will be crucial to express the full potential of precision oncology in ovarian cancer.

## Figures and Tables

**Table 1:** Indications for risk-reducing surgery.

	BRCA1	BRCA2	PVs OF OTHER GENES	LYNCH SYNDROME
ESMO	RRBSO between ages 35 and 40	RRBSO between ages 40 and 45	BRIP1, RAD51C or RAD51D: RRBSO should be considered at age 45–50 PALB2: RRBSO may be considered in post menopausal women	Prophylactic hysterectomy + BSO should be discussed in women who have completed childbearing or are postmenopausal
NICE	RRBSO no earlier than 35 years	RRBSO no earlier than 40 years	RRBSO no earlier than 45 years	Prophylactic hysterectomy + BSO no earlier than 35 years
NCCN (including Middle East and North Africa adaptation)	RRBSO between ages 35 and 40 Hysterectomy could be discussed	RRBSO between ages 40 and 45 Hysterectomy could be discussed	BRIP1, PALB2, RAD51C, RAD51D: RRSO starting at age 45–50 years	Prophylactic hysterectomy + BSO may be considered starting at age 40 years

PV: pathological variants; RRBSO: risk-reducing bilateral salpingo-oophorectomy.

**Table 2:** (**A**) Comparison of surgical management of epithelial ovarian cancer. (**B**) Comparison of surgical management of high-grade serous ovarian cancer.

	ESMO-ESGO	NCCN	NICE	BGCS	JSGO
**(A)**					
STIC	Peritoneal staging if positive peritoneal cytology. TAH should be considered (++ if BRCAm) or alternatively endometrial sampling. Routine LN not mandatory. CHT not recommended	Option 1: OBS Option 2: surgical staging followed by obs or CHT if invasive disease		If peritoneal cytology negative and imaging negative: completion with bilateral oophorectomy. If peritoneal cytology positive: surgical staging	
LGSC	Removal of all visible peritoneal implants + formal peritoneal staging + PDS. NACT + IDS can be considered. Routine LN not recommended	PDS. NACT + IDS not recommended.		PDS	
Special subtypes	TAH + BSO + OMT + systematic pelvic and para-aortic LN + peritoneal biopsies	Comprehensive surgery + systematic LN. Appendicectomy is recommended in MOC		Comprehensive surgery. LN may be omitted in low-risk subtypes. Appendicectomy may be considered.	
BOT	TAH not mentioned. BSO in menopausal women. Appendicectomy not recommended. LN not recommended	TAH + BSO and debulking as needed. LN and OMT not strictly recommended		Routine TAH not recommended. Appendicectomy only if appendix is pathological. Early-stage serous BOT: bilateral cystectomy. Restaging surgery (ipsilateral oophorectomy + peritoneal cytology + peritoneal biopsies + omentectomy) if micropapillary features. Early-stage mucinous BOT: unilateral adnexectomy BSO in post-menopausal women Advanced stages: debulking surgery. LN not recommended	TAH + BSO + OMT + peritoneal cytology + biopsies
**(B)**					
HGSC I–II	TAH + BSO + peritoneal washing or cytology prior to manipulation of tumor is the standard + peritoneal biopsies + at least infracolic OMT + bilateral pelvic and para-aortic LN	TAH + BSO + OMT + peritoneal cytologic examinations (ascites or peritoneal lavage) + random peritoneal biopsies from pelvic, paracolic gutters and undersurfaces of diaphragm (alternatively, PAP scrap on diaphragm) or biopsies of any suspicious lesions.	TAH + BSO + infracolic OMT + biopsies of any peritoneal deposits + random biopsies of the pelvic and abdominal peritoneum + LN	TAH + BSO + peritoneal washing/ascitic sampling taken before manipulation, peritoneal biopsies and omental biopsies/OMT	TAH + BSO + OMT + pelvic/para-aortic LN + peritoneal cytology + biopsies from sites in the abdominal cavity. Moreover, specimens are suggested to be acquired from the surface of the pouch of Douglas, abdominal wall, diaphragm, bowel, and mesentery, in addition to the suspected lesions
HGSC III–IV	Maximal surgical effort with no macroscopical residual disease	Maximal surgical effort with no macroscopical residual disease	Maximal surgical effort with no macroscopical residual disease	Maximal surgical effort with no macroscopical residual disease	Maximal surgical effort with no macroscopical residual disease
NACT + IDS	Can be considered. HIPEC during IDS not a standard therapy	Can be considered. HIPEC during IDS can be considered	Can be considered.	Can be considered. HIPEC during IDS can be considered in specialized centers	Can be considered.
MIS approach	Only in early stages when performed by gyn-onco surgeon	Only in early stages, when RT = 0 is achievable and by a gyn-onco surgeon			Not recommended
Lymphadenectomy	Recommended in early stages. Not routinely recommended in advanced stages, removal only if suspicious	Stage IA-IIA: recommended Stage IIB or more: not recommended, removal only if suspicious	Recommended in early stages. Not routinely recommended in advanced stages	Recommended in early stages in absence of peritoneal dissemination. Not routinely recommended in advanced stages	Stage IA-IIA: recommended Stage IIB or more: not recommended, removal only if suspicious

STIC = serous tubal intraepithelial cancer; LN = lymphadenectomy; OBS = observation; CHT = chemotherapy; TAH = total abdominal hysterectomy; BSO = bilateral salpingo-oophorectomy; OMT = omentectomy; PDS = primary debulking surgery; IDS = interval debulking surgery; NACT = neoadjuvant chemotherapy; BOT = borderline ovarian tumor; MOC = mucinous ovarian cancer. HGSC = high-grade serous cancer; MIS = minimally invasive surgery; HIPEC: Hyperthermic Intraperitoneal Chemotherapy; RT = residual tumor.

**Table 3:** Systemic therapy in Borderline Ovarian Tumor and Low Grade Serous Ovarian Cancer.

Cancer Type	BGCS	NCCN	ESMO-ESGO	ESMO	JSGO	Australian Guidelines
	Recommended Options					
BOT	Not recommended	Adjuvant CT may be considered if invasive implants			Adjuvant CT may be considered if invasive implants	Adjuvant CT may be considered if invasive implants
LGSOC	Carboplatin-paclitaxel in advanced disease Endocrine therapy or Trametinib in recurrent setting; other targeted therapies can be considered depending on molecular profile	Carboplatin-paclitaxel, ± maintenance letrozole or other hormonal therapy in stages II-IV (may be considered in stage IC) Endocrine therapy or Trametinib in recurrent setting	Carboplatin-paclitaxel ± bevacizumab in stage > I Maintenance therapy can be considered in stages III–IV Endocrine therapy or Trametinib in recurrent setting	Carboplatin-paclitaxel in stages II–IV (optional in stages IB–IC)		

BOT = Borderline ovarian tumor; LGSOC: low-grade serous ovarian cancer; CT = chemotherapy.

**Table 4:** Systemic therapy in early stages of epithelial ovarian cancer.

	BGCS	NCCN	ESMO-ESGO	ESMO	JSGO	Australian
	Recommended Options					
Regimen	Carboplatin/paclitaxel or platinum alone	Carboplatin/paclitaxel or carboplatin/PLD or Docetaxel/carboplatin		Carboplatin/paclitaxel		Platinum compound
Number of cycles	6 cycles; 3 cycles are appropriate for non-serous hystotype			6 cycles; 3 cycles are appropriate for non-high-grade OC		
HGSOC	Recommended	Recommended	Recommended	Recommended	Recommended	Recommended
Endometrioid OC	Can be omitted in IA G1-G2			Can be omitted in IA low grade; optional in IB–IC low grade	Can be omitted if IA–IB low grade	Can be omitted if IA–IB low grade
CCC	Can be considered		Can be omitted in IA–IB; optional in IC1	Optional for stages < IC1	Recommended	
Mucinous OC	Can be omitted in IA expansile G1-G2	Can be omitted in IA–IB expansile; optional in IC expansile and IA infiltrative		Can be omitted in IA–IB expansile; optional in IC expansile and IA infiltrative	Can be omitted if IA–IB low grade	

OC = ovarian cancer; HGSOC = high-grade serous ovarian cancer; CCC = clear cell ovarian cancer; PLD = pegylated liposomal doxorubicin.

**Table 5:** Systemic therapy in advanced stages of epithelial ovarian cancer.

	BGCS	NCCN	ESMO-ESGO	ESMO	JSGO	Australian
	Recommended Options					
Regimen	Carboplatin AUC 5–6 and paclitaxel 175 mg/m^2^ (i.v.) every 3 weeks	Carboplatin AUC 5–6 and paclitaxel 175 mg/m^2^ (i.v.) every 3 weeks		Carboplatin AUC 5–6 and paclitaxel 175 mg/m^2^ (i.v.) every 3 weeks	Carboplatin and paclitaxel	Platinum compound
Alternatives	paclitaxel can be replaced by docetaxel or pegylated liposomal doxorubicin	Docetaxel/carboplatin or Carboplatin/liposomal doxorubicin or Paclitaxel weekly/carboplatin every 3 weeks		Docetaxel/carboplatin or Carboplatin/liposomal doxorubicin	Docetaxel/carboplatin or Carboplatin/liposomal doxorubicin	
Addition of Bevacizumab	Can be considered in stage III and macroscopic disease or stage IV	Can be considered in NACT	Recommended	Should be considered	Recommended	Can be considered in stage III and macroscopic disease or stage IV
Number of cycles	6 cycles	6 cycles, at least 3 adjuvant cycles		6 cycles		

AUC = area under curve; i.v. = intravenous; NACT = neoadjuvant chemotherapy.

**Table 6:** Systemic therapy in recurrence setting.

	BGSC	NCCN	ESMO-ESGO	ESMO 2023	JSGO
	Recommended Options				
Platinum sensitive	Platinum rechallenge	Platinum rechallenge	Platinum rechallenge If intolerant: trabectedin-PLD	Platinum rechallenge If intolerant: trabectedin-PLD	Platinum rechallenge
Platinum resistant	Non-platinum single agent	Non platinum-agents	Non-platinum single agent Supportive care	Non-platinum single agent Supportive care	Non-platinum single agent
Bevacizumab	May be considered in platinum-resistant patients	Alone or in combination in both platinum-sensitive and -resistant settings As single-agent maintenance	May be considered if already received in 1st line May be considered in platinum-resistant patients BRCAwt/unknown: Recommended as maintenance if not received in 1st line	Alone or in combination in both platinum-sensitive and resistant settings As single-agent maintenance	Recommended for platinum-resistant patients Consider as maintenance therapy if previous CT + Bevacizumab
PARP maintenance	Can be considered if not received in 1st line	Can be considered if not prior progression on PARPi	BRCAmut: Recommended if not received in 1st line BRCAwt/unknown: Recommended if not received in 1st line PARPi rechallenge	Recommended for platinum-sensitive patients	Can be considered

PLD = pegylated liposomal doxorubicin; BRCAmut = BRCA mutated; BRCAwt = BRCA wild type; PARP = Poly (ADP-ribose) Polymerase; CT = chemotherapy.

## Data Availability

All data relevant to the study are included in the article or uploaded as Supplementary Information. All data were extracted from previously published studies; thus, they are publicly available.

## Supplementary Materials

The following supporting information can be downloaded at: https://www.mdpi.com/article/10.3390/cancers17243915/s1, Table S1: Surveillance protocols; Table S2: Clinical examination according to guidelines; Table S3: Use of ultrasound according to guidelines; Table S4: Use of advanced imaging according to guidelines; Table S5: Use of tumor markers according to guidelines; Table S6: Pre-operative workup; Table S7: Indications for maintenance treatment; Table S8: Use of radiotherapy according to different situations; Table S9: Fertility sparing surgery recommendations; Table S10: Follow-up strategies across guidelines; Table S11: Utility and limitations of CA125 dosage according to guidelines.
