# Supplementary material for: Management of Patients with Epithelial Ovarian Cancer: A Systematic Comparison of International Guidelines from Scientific Societies (AIOM-BGCS-ESGO-ESMO-JGSO-NCCN-NICE)
Source: Cancers (Basel). 2025 Dec 7;17(24):3915. doi: 10.3390/cancers17243915 (PMC12730224; doi:10.3390/cancers17243915)
Supplement: Supplementary file 1 [file cancers-17-03915-s001.zip › cancers-3993698-supplementary.pdf]

Management of Patients with Epithelial Ovarian Cancer: A Systematic Comparison of International Guidelines from Scientific Societies (AIOM – BGCS - ESGO - ESMO – JGSO – NCCN - NICE)

Supplemental material

Table S1. Surveillance protocols.

	BRCA1	BRCA2	PVs OF OTHER GENES	LYNCH SYNDROME
**ESMO**	6-monthly TVUS + serum CA125 from the age at which BSO is recommended until RRS	6-monthly TVUS + serum CA125 from the age at which BSO is recommended until RRS		Yearly TVUS + serum CA125 from age 30-35
**NICE**	serial 4-monthly CA125 longitudinal testing using an algorithm with demonstrated accuracy (for example, the Risk of Ovarian Cancer Algorithm Test)			
**AUSTRALIAN GUIDELINES**	TVUS + serum CA125 is not recommended			
**NCCN**	No data supporting surveillance protocols			

TVUS: transvaginal ultrasound; RRS: risk reducing surgery; BSO: bilateral salpingo-oophorectomy; RRS: risk reducing surgery.

Table S2. Clinical examination according to guidelines.

Guidelines	Recommended Clinical Assessment
**ESMO-ESGO**	Detailed history (bloating, pelvic pain, early satiety, bowel/urinary symptoms), full physical exam (abdomen, pelvis, rectum, nodes)
**BGCS**	Same as ESGO/ESMO + consider age, family/personal history, ascites or fixed masses; combines with CA-125 in women > 50.
**AIOM**	Assumes proper initial assessment; no detailed recommendations
**NCCN Africa**	Focused clinical history, bimanual exam, abdominal palpation for ascites, family and nutritional history.

Table S3. Use of ultrasound according to guidelines.

Guidelines	Ultrasound Recommendation	Use of Models
**ESMO-ESGO**	First-line tool (TSV and TAS)	Supports IOTA models, esp. ADNEX
**BGCS/NICE**	TVS + CA-125 if symptoms suggestive of cancer	Favors ADNEX over MRI
**NCCN Africa**	TVS/TAS essential even il low-resource setting	Structured criteria (bilaterality, ascites, ect..)
**AIOM**	Not specified	Assumes imaging pre-staging

TSV = Trans Vaginal Sonography; TAS = Trans Abdominal Sonography.

Table S4. Use of advanced imaging according to guidelines.

Guideline	CT	MRI	PET-TC
**ESMO-ESGO**	Fist-line for staging	Second-level (young women, indeterminate masses)	Not routine; useful for extraoperitoneal lesions
**BGCS**	Fist-line for staging	Second-level (young women, indeterminate masses)	Selective use

**NCCN Africa**	CT when avaible; MRI if CT inconclusive	MRI preferred when needed	Use only if results will change management

CT= computed tomography; MRI = Magnetic Resonance Imaging; PET = positron-emission tomography.

Table S5. Use of tumor markers according to guidelines.

Marker	ESMO-ESGO	BGCS	NCCN Africa
CA-125	Referenced marker	Used routinely	Used with caution
HE4	Complement to CA-125 in selected cases	Not recommended routinely	Considered in some settings
CEA/CA19.9	Used to rule out GI origin (esp. mucinous)	Used to rule out GI origin (esp. mucinous)	Not emphasized

Table S6. Pre-operative work up.

	ESMO-ESGO	NCCN	NICE	BGCS	JSGO
**Preoperative imaging**	Chest-abdomen-pelvis CT scan	Chest-abdomen-pelvis CT scan		Chest-abdomen-pelvis CT scan	
**Laparoscopic pre-operative assessment**	Recommended	Recommended		Recommended	Recommended
**Molecular testing**	High grade non mucinous EOC: BRCA and HRD testing; endometrioid and clear cells carcinomas: MMR testing	Ovarian cancer: BRCA and HRD testing	High grade non mucinous EOC: germline genetic testing	High grade non mucinous EOC: BRCA and HRD testing; endometrioid and clear cells carcinomas: MMR testing	

CT= computed tomography; HRD= homologous recombination deficiency; MMR= missmatch repair; EOC=epithelial ovarian cancer.

Table S7. Indications for maintenance treatment.

	BGSC	NCCN	ESMO-ESGO	ESMO 2023	JSGO
**Recommended options**					
**Bevacizumab**	Recommended	Recommended<br>BRCAwt<br>BRCA status unknown	Recommended<br>BRCAwt<br>HRD (-)<br>BRCA status unknown	Recommended<br>HRD (-)	Recommended
**Bevacizumab + Olaparib**	BRCAmut<br>HRD (+)	Regardless of mutational status		BRCAmut<br>BRCAWT<br>HRD (+)	
**Olaparib**		BRCAmut		BRCAmut	BRCAmut
**Niraparib**		BRCAmut (if bevacizumab in first line)<br>BRCAwt<br>BRCA status uknown	BRCAwt<br>HRD (-)<br>BRCA status unknown	BRCAmut<br>BRCAwt<br>HRD (+)<br>HRD (-)	
**Rucaparib**			BRCAwt<br>HRD (-)		

	BRCA status unknown	
**Duration**	Bevacizumab: 15 months<br>Olaparib: 2 years<br>Niraparib: 3 years<br>Rucaparib: 2 years	Bevacizumab +Olaparib: 2 years<br>Olaparib: 2 years<br>Niraparib: 3 years

HRD = Homologous Recombination deficient; BRCAmut = BRCA mutated; BRCAwt = BRCA wild type.

Table S8. use of radiotherapy according to different settings.

Clinical scenario	Main recommendations
Adjuvant after initial surgery	RT is not standard; WAI excluded except in historical or selected contexts
Widespread systemic recurrence	RT is not recommended may be considered for local control selected patient
Oligorecurrence/single metastasis	SBRT or stereotactic RT may be considered for local control selected patients
Palliative setting (localized sympyoms)	RT can be used to relieve symptoms (pain, compression)

RT = radiotherapy; SBRT = Stereotactic Body Radiotherapy; WAI = Whole Abdomen Irradiation.

Table S9. Fertility sparing surgery recommendations.

Guidelines	Tumor considered for FSS	Stage	Main Notes
**AIOM**	BOT, epithelial G1-G2 OC	FIGO 1	Preservation of uterus + 1 ovary, full staging; lymphadenectomy not recommended
**BGCS**	BOT, favorable epithelial G1-G2 OC	IA-IC	Individualized approach via MDT; regular ultrasound follow-up.
**ESMO-ESGO**	BOT, low-grade epithelial OC	IA-IC (selected IC2)	Oncofertility centers; use of IOTA adenex diagnostic models
**ESMO**	Low-grade epithelial OC (serous, endometrioid, mucinous)	I-II	Only after full staging and no-extra ovarian involvement
**NCCN**	BOT, epithelial IA-IC1, mucinous	IA-IC1, IB (uterus only)	FSS not recommended for clear cell carcinoma
**JSGO**	Non-clear-cell epithelial OC	IA-IC1	For clear cell, only IA; USO + multiple biopsie in BOT
**SUB-SAHARIAN AFRICA ADAPTATION**	BOT, low-risk epithelial OC	Early stage	Minimally invasive approach in selected cases
**AUSTRALIAN GUIDELINES**	Any hystology		Supports preservation techniques, without specific guidance on FSS

FSS = fertility sparing surgery; BOT = borderline ovarian tumor; MDT = multidisciplinary team; USO = unilateral salpingo-oophorectomy; OC = ovarian cancer.

Table S10. Follow up strategies across guidelines.

Guideline	First 2 years	Years 3–5	After 5 years
**NCCN**	Every 2–4 months	Every 3–6 months	Annually
**BGCS**	Every 3 months	Every 6 months	After 5 years: individualized
**ESM-ESGO**	Every 3–4 months	Every 6 months	>5 years: individualized
**JSGO**	Every 2–4 months	Every 3–6 months	Annually
**AUSTRALIAN GUIDELINES**	Every 3 months	Every 4–6 months	Annually
**AIOM**	Not rigidly defined	Individualized	--

Table S11. Utility and limitation of CA125 dosage according to guidelines.

Guidelines	Utility	Limitations	Therapeutic Implication
**BGCS**	Can be monitored, but with caution.	MRC/EORTC trial showed no survival benefit from treating at early biochemical relapse; early treatment worsened global health scores	Do not initiate chemotherapy based only on CA-125 increase. Wait for symptoms or radiologic confirmation
**NCCN**	Useful if elevated at diagnosis.	Possible anxiety from repeated testing; limited standalone value.	Treatment should not be started based solely on CA-125; requires clinical or radiographic evidence.
**JSGO**	Useful when elevated at baseline.	Not recommended as the sole determinant for therapeutic decisions.	Clinical and/or radiological confirmation required before treatment.
**AUSTRALIAN GUIDELINES**	Valuable when initially elevated; supports follow-up	Should not guide treatment decisions on its own.	Clinical and radiologic confirmation essential prior to intervention.
**SUB-SAHARIAN AFRICA ADAPTATION**	Recommended only if CA-125 was initially elevated.	Similar to NCCN: patient anxiety; uncertain benefit in the era of advanced imaging.	Similar to NCCN: patient anxiety; uncertain benefit in the era of advanced imaging
